# Extracellular vesicles from medicated plasma of Buyang Huanwu decoction-preconditioned neural stem cells accelerate neurological recovery following ischemic stroke

**DOI:** 10.3389/fcell.2023.1096329

**Published:** 2023-03-01

**Authors:** Jun Long, Chenyang Gu, Qiankun Zhang, Jiale Liu, Jiajun Huang, Yajing Li, Yifan Zhang, Rong Li, Waqas Ahmed, Jianfeng Zhang, Ahsan Ali Khan, Hengsen Cai, Yong Hu, Lukui Chen

**Affiliations:** ^1^ Department of Neurosurgery, Neuroscience Center, Integrated Hospital of Traditional Chinese Medicine, Southern Medical University, Guangzhou, China; ^2^ Affiliated Dongguan Hospital, Southern Medical University (Dongguan People’s Hospital), Guangzhou, China; ^3^ Department of Neurology, Affiliated Zhongda Hospital, School of Medicine, Southeast University, Nanjing, China; ^4^ Section of Neurosurgery, The Aga Khan University, Karachi, Pakistan; ^5^ Department of Neurosurgery, The Second People’s Hospital of Pingnan, Pingnan, China; ^6^ Department of Orthopedics and Traumatology, The University of Hong Kong, Hongkong SAR, China

**Keywords:** ischemic stroke, neural stem cells, extracellular vesicles, Buyang Huanwu decoction, medicated plasma, neurogenesis, miRNAs profiles

## Abstract

**Introduction:** The neurological impairment of survivors after ischemic stroke poses a serious risk to their quality of life and health. Effective therapeutic options are still lacking. Neural stem cells (NSCs) promote neurogenesis *via* secreted extracellular vesicles (NSC-EVs), which would be a potential therapeutic option, but the insufficient quantity of NSC-EVs *in vivo* restrains clinical application. Buyang Huanwu Decoction (BHD), a classic traditional Chinese medicine (TCM) decoction, is promising to alleviate neurological impairment after ischemic stroke. It was speculated that BHD might promote neurological recovery through the NSC-EVs.

**Methods:** The medicated plasma of BHD (MP-BHD) was prepared to precondition NSCs and isolate EVs (BHD-NSC-EVs). Middle cerebral artery occlusion (MCAO) models and primary NSCs were administered to evaluate the therapeutic effect. Next-generation sequencing was performed to explore the mechanism.

**Results:** The BHD-NSC-EVs more significantly accelerated neurological recovery after MCAO and promoted NSCs proliferation and differentiation than BHD and NSC-EVs alone. MP-BHD enhanced the largescale generation of BHD-NSC-EVs, which encapsulated functional miRNA and may play critical roles in neurogenesis.

**Discussion:** In replacing BHD or NSCs, the preconditioned NSC-EVs present a more efficient therapeutic strategy for ischemic stroke. Based on the clinical efficacy of TCM, the preconditioning of NSC-derived EVs *via* the MP of TCM herbs would presents a newly promising therapeutic strategy for neurological diseases.

## 1 Introduction

The neurological impairment of the survivors of an ischemic stroke, especially severe limb motor dysfunction (e.g., partial paralysis), poses a serious risk to the quality of life and health of the patients (Coscia et al., 2019; Lin et al., 2019). According to the pathology, this form of neurological impairment involves losses of neurons, damages of axons and a reduction in blood supply, meaning promoting neurovascular unit regeneration is a promising therapeutic strategy for enhancing stroke recovery and improving the long-term outcomes (Ozaki et al., 2019). However, specific and effective therapeutic options are still lacking.

Neural stem cells (NSCs) reside in neurogenic niches, such as the subgranular zone and subventricular zone of the adult brain, and have the ability to self-renew and the potential to differentiate into other cell lines, including neurons, oligodendrocytes, and astrocytes (Llorente et al., 2022). Hence, the adoption of NSCs transplantation to replace lost cells and promote structural repair in cerebral infarction presents an ideal therapeutic strategy (Russo et al., 2018; Lyu et al., 2021). However, it is challenging to directly transplant extrinsic NSCs into the target tissues due to the presence of ischemia, the low survival rate of the transplanted stem cells, cell de-differentiation, tumor formation, and immune rejection, which further limits the clinical application of direct NSC transplantation for post-ischemic-stroke treatment (Zhao et al., 2022).

NSCs induce neuroprotection and promote repair through a paracrine mechanism that secretes small extracellular vehicles (EVs) (Contreras et al., 2022), which play an important role in these processes. The NSC-EVs with a diameter of 50–200 nm are the small endocytic membrane-bound nanovesicles that hold cargos, including proteins, coding and non-coding RNAs, and DNAs, and can mirror the functions of original NSCs and play crucial roles in intercellular communication. Compared with direct NSC transplantation, NSC-EVs have many advantages, such as low immunogenicity, good biodegradability, the ability to encapsulate endogenous bioactive molecules, and the ability to cross the blood–brain barrier (Zhang et al., 2021a; Li et al., 2021). However, the technical challenge of producing sufficient quantities limits the application of NSC-EVs in neuroprotection and neurological functional repair *in vivo* (Stewart et al., 2016; Reed and Escayg, 2021).

Buyang Huanwu Decoction (BHD), which was created and prescribed by Wang Qingren, a physician of the Qing dynasty in ancient China, is a famous herbal formula used for neurological defect-related sequelae of cerebral ischemia and has been widely applied in clinical practice (Zheng et al., 2021). The effects of promoting the recovery of the motor, sensory, and/or autonomic function of the survivors of an ischemic stroke has been widely verified by evidence-based research (Gao et al., 2021; Nie et al., 2021) and an increasing number of patients have benefited from the treatment. However, the underlying molecular mechanisms have not, as yet, been elucidated in detail.

Mounting evidences suggest that BHD exerts a therapeutic effect on ischemic stroke patients through inducing nerve regeneration and promoting angiogenesis (Lee et al., 2020; Zhuge et al., 2020). It was speculated that the process of inducing the nerve regeneration *via* BHD may be involved in the activation of intrinsic NSCs, enhancing the proliferation, migration, differentiation, and integration of NSCs from neurogenic regions to the infarcted lesion. Since NSC-EVs contain cargos involved in the rapid signaling transduction that affects the activity of the recipient cells, which is more beneficial for nerve regeneration and repair than NSCs alone, it is believed that NSC-EVs play a key role in the neuroprotection and repair efficacy of NSCs. Furthermore, BHD may promote the NSC-EV paracrine mechanism to activate intrinsic NSCs to promote the recovery of neurological functioning.

To test the above hypotheses, in this study, the medicated plasma of BHD (MP-BHD) was prepared to precondition primary NSCs and the corresponding EVs (BHD-NSC-EVs) were isolated to evaluate the therapeutic efficacy of BHD-NSC-EVs on neurological impairment recovery following ischemic stroke though middle cerebral artery occlusion (MCAO) model rats and primary NSCs.

## 2 Materials and methods

### 2.1 Preparation of BHD and MP-BHD

BHD contains Huang-Qi (Radix Astragali), Chi-Shao (Radix Paeoniae Rubra), Dang-Gui (Radix Angelicae Sinensis), Chuan-Xiong (Rhizoma Chuanxiong), Tao-Ren (Semen Persicae), Hong-Hua (Flos Carthami), and Di-Long (Lumbricus). These herbal pieces were provided by the Department of Pharmacy of TCM-Integrated hospital. According to the methods described in Pharmacopoeia of China, all herbal pieces above were mixed at a ratio of 60:6:4.5:3:3:3:3 (dry weight), then were chopped, immersed in distilled water for 1 h and boiled for 30 min at 100°C. After twice cooking in above way, the decoctions were mixed and concentrated to a decoction containing raw medicine 1 g/mL and refrigerated for later use.

BHD 25.8 g·kg^−1^·d^−1^, which drug dosage is calculated according to body surface area and is twice the clinical equivalent adult human dose of 12.87 g·kg^−1^·d^−1^, was intragastrically administrated twice per day for 7 days according to previous reports (Zhou et al., 2012). The dosages of BHD in clinical practice was defined as effective dose. After the rats were fasted for 12 h and received the last administration of BHD for 120 min, arterial blood was collected in a tube of anticoagulant, mixed gently, and plasma was collected by the sobbin method. 40 mg sobbin and 0.5 mL adsorption buffer were added into 1 mL plasma, fully mixing, shake well on the gyromixer, and shake slowly for 10 min. After centrifugation at 5,000 r/min for 15 min, the supernatant treated with soapy soil adsorption once as above. Remove fibrinogen to obtain plasma. Medicated plasma containing BHD was defined as MP-BHD.

### 2.2 Extraction, culture, and identification of NSCs

From SD rat embryos at 13.5 days of gestation, the hippocampal tissue was mechanically isolated in ice-cold phosphate buffer saline (PBS), and the cells were collected by centrifugation and resuspended in dulbecco’s modified eagle medium (DMEM)/F12 medium with 2% B27 (Gibco, Grand Island, NY, United States), bFGF 20 ng/mL (Novoprotein Scientific Inc., Suzhou, China), and EGF 20 ng/mL (Sino Biological Inc., Beijing, China), glutamine (2 mmol/L), 1% penicillin/streptomycin. The cells were passed to the third generation for experiment.

NSCs were planted on poly-lysine-coated glass slides for cell adhesion, which were fixed with paraformaldehyde (PFA, 4%, w/v, pH 7.4), permeated with 0.3% Triton X-100, and blocked with goat serum. The NSC spheres consisting of NSCs were labeled with the NSCs markers sex determining region Y-box 2 (SOX2) and Nestin antibody at 4°C overnight, and then treated with fluorescent secondary antibody at room temperature for 2 h. The nuclei were stained with 4′, 6-diamidino-2-phenylindole (DAPI), and the plates were sealed and fixed, and detected by immunofluorescence (IF) staining with fluorescence microscopy.

### 2.3 Isolation and identification of EVs

EVs were isolated from the serum-free culture of NSCs. Briefly, 6 × 10^6^ NSCs were plated on 10 cm dish and cultured in 10 mL NSC proliferation medium. The final concentration of MP-BHD in medium is 10% of the reaction system, which was defined as MP-BHD-M (MP-BHD-L and MP-BHD-H is 5% and 20% respectively), the control group were treated with PBS. After 12 h of treatment, the supernatant was collected and filtered through a 0.22 μm filter, centrifuged at 2000 g for 10 min at 4°C, centrifuged at 10,000 g for 1 h at 4°C, ultracentrifuged at 150,000 g for 1.5 h at 4°C. After centrifugation, the supernatant was removed, and The bottom of the centrifuge tube was resuspended with 100 μL PBS to precipitate and stored in the refrigerator at −80°C. The EVs from the NSCs treated with PBS, OGD, Heat and MP-BHD, was defined as PBS-NSC-EVs, OGD-NSC-EVs, Heat-NSC-EVs, and BHD-NSC-EVs respectively.

### 2.4 Nanoparticle tracking analysis (NTA)

NanoSight LM10 instrument (Malvern Instruments Ltd., Malvern, United Kingdom) was used to determine the size distribution and quantity of NSC-EVs and BHD-NSC-EVs. EVs samples were diluted ten times with PBS to reach optimal concentration for instrument linearity. Data were analyzed with the NTA software version 3.1.54.

### 2.5 Transmission electron microscopy (TEM)

The morphology of the obtained EVs was observed directly by TEM (Hitachi H7500, Tokyo, Japan) to measure the diameter of EVs. Before observation, 3 μL of EVs samples was placed on formvar carbon-coated 200-mesh copper electron microscopy grids and incubated for 5 min at room temperature. Following, the EVS samples was subjected to standard uranyl acetate staining.

### 2.6 Western blotting (WB) for EVs surface markers

WB was used to detect the specific EV surface markers CD81, CD9, CD63, Calnexin and ALIX, and TSG101 (The supernatant of NSCs was collected after the third passage). The total RNA concentration and purity were detected using Nanodrop 2000 (Thermo Fisher Scientific, United States). The total protein concentration of EVs was determined using a bicinchoninic acid assay (BCA, Thermo Fisher Scientific, Waltham, MA) by absorbance (OD value) at 562 nm.

### 2.7 Preparation of MCAO model and evaluation

110 adult male specific-pathogen free SD rats, aging 2.5–3 months, weighing 250–300 g, were supplied by Animals Laboratory Centre of Southeast University. Rats were housed in the environment of 50% humidity and 24°C temperature, 12 h dark and light cycle, and were received free access to diet and water.

The rats were deprived of water before the establishment of left MCAO model. After the rat was anaesthetized with isoflurane (RWD Inc., Guangzhou, China) (3% induction, 1.5% maintenance) in oxygen (0.4 L/min) and nitrogen (0.6 L/min) and routinely disinfected. The common carotid artery (CCA), external carotid artery (ECA) and internal carotid artery (ICA) were isolated and exposed fully. The embolization line was inserted into ICA *via* ECA, and reperfusion was performed at 90 min after ischemia, the ECA was ligated, after which the wound was sutured. Sham operation group was treated with the same operation method, but only the neck artery group was isolated without embolization line.

24 h after operation, neurological function score was performed to determine whether the MCAO model was successful or not. Zea Longa 5-point scale was used to assess the model. The left front paw could not stretch for 1 point; Walk to the left turn circle is 2 points; The left side of the line is 3 points; Loss of spontaneous activity and consciousness were 4 points. The final score of the rats after modeling was 1-3, indicating the success of modeling and entering the next stage of the experiment. The rats with scores of 0 and 4 failed to build the model and withdrew from the experiment.

### 2.8 TTC staining

To evaluate the effect of MCAO surgery, 2, 3 , 5-triphenyl tetrazolium chloride (TTC; Sigma-Aldrich) staining was used 24 h after MCAO. The brain was cut into six consecutive coronal sections, 2% solution of TTC for 30 min at 37°C in dark place. The infarct volumes were calculated with Image-J analysis software (Media Cybernetics, Silver Spring, MD, United States).

### 2.9 Group design and drug administration

After operation, the rats were randomly divided into five groups: Sham group, in which the rats were treated with nothing; PBS group, in which the MCAO model rats were given tail-intravenous injection of PBS 0.1 mL; BHD group, in which the MCAO model rats were given intragastric administration of BHD at a dose of 25.8 g·kg^−1^·d^−1^, twice a day; NSC-EVs group and BHD-NSC-EVs group, in which the rats were given tail-intravenous injection of NSC-EVs and BHD- NSC-EVs 0.1 mL (100 µg/100 µL) once a day, respectively. Meanwhile the rats were intraperitoneally injected with BrdU (50 mg/kg), once a day. All rats were treated for 28 consecutive days and were allowed to move and access food and water freely.

### 2.10 Animal behavior test

#### 2.10.1 Footprint analysis

Gait and motor coordination were evaluated in 28th day after MCAO model. The front and rear paws were coated with dyes of red and green colors. A rat was placed on a piece of absorbent paper surrounded by a cage to encourage it to walk in a straight line. Then, the footprint was digitized and the representative picture was used to assess coordination.

#### 2.10.2 CatWalk gait analysis

The gait parameters of the rats were collected 1 day after operation and intervened for 28 days. The rats were allowed to enter the CatWalk runway smoothly, and the rats were allowed to traverse the runway spontaneously and without stopping under any stimulus. The test rats passed the runway 3 times without interruption and at a roughly uniform speed within 5 s, and the total number of single steps in the collection range was not less than six steps. The speed of walking was recorded.

#### 2.10.3 Rotating-rod walking test

The experimental score of walking on the rotary rod: 0: the rats could walk on the rod during the rotating process; 1 point: the rat will not fall down during the rotation, and the time is over 60 s; 2 points: the rat fell off the stick after the rotation began; 3 points: The rat falls off the stick before the rotation begins.

#### 2.10.4 Montoya’s staircase test

The rats were placed in a transparent box made of plexigla, and there were seven stairs on both sides of the box. 45 mg food pellets were placed in the circular trough of each staircase, and added once every 5 min. The number of food pellets grasped by the left forelimb within 1 h was used as the score of the motor function of the forelimb. The left forelimb motor function was trained 2 weeks before cerebral ischemia/reperfusion (I/R), and the left forelimb motor function was evaluated 1 and 2 weeks after cerebral I/R.

#### 2.10.5 Myodynamia of front paws

Let the forelimb of the rat grasp the metal triangle rod, and slowly drag the tail of the rat backward. When the forelimb of the rat released, the highest value was recorded as the maximum grip. Each rat was measured 5 times, and the average value was calculated for statistical analysis.

### 2.11 Magnetic resonance imaging (MRI)

An anesthetized rat was placed prone on the fixation system and examined using a small animal MRI system (Bruker BioSpec7T/20 USR; Bruker AXS GmbH, Karlsruhe, Germany). T2-weighted images were obtained in coronal plane with ParaVision (version 6.0.1, Bruker BioSpec; Bruker AXS GmbH). The sequence protocol was executed with the following parameters: T2-weighted; 256 × 256 matrix; slice thickness, 1 mm; intersection gap, 1 mm; echo time/repetition time: 27/3,000 ms; rapid acquisition with relaxation enhancement factor, 16; flip angle, 90°.

### 2.12 Laser speckle imaging system (LSIS)

The blood perfusion volume and vessel diameter were measured by LSIS. The skin was cut through the midsagittal line of the cranial apex and the serosal membrane on the skull surface was peeled off. Two drops of bupivacaine hydrochloride were added for local analgesia, and then the skull was polished with a skull drill until the angiography was clear. Adjust the focal length, false color threshold and magnification parameters. Optical imaging was performed again at first, seventh, 14th, 28th day after MCAO and treatment. The cerebral blood flow perfusion volume and vessel diameter were extracted from the image information offline, and the percentage values of cerebral ischemia and I/R were calculated according to the basic values.

### 2.13 BHD-NSC-EVs uptake *in vivo* and *in vitro*

To monitor the biodistribution of BHD-NSC-EVs *in vivo*, EVs were fluorescent labelled, 4 mg/mL DiR (1,1-dioctadecyl-3,3,3,3-tetramethy-lindotricarbocyanine iodide; Umibio Group, Shanghai, China) solution was added to PBS (1:200) and incubated according to the instructions of manufacturer. Excess dye from labeled EVs was removed by centrifugation at ×10,000 g for 30 min at 4°C. Then, isolated EVs were washed three times by resuspending the pellet in PBS after ultracentrifugation at ×100,000 g for 1 h at 4°C. The final pellet was resuspended in PBS, and the supernatant was also collected as the control.

0.1 mL (100 µg/100 µL) DiR-EVs were injected into the MCAO model rats with tail vein injection. After 6 h or 12 h later, the rats were anesthetized, the images of BHD-NSC-EVs distribution *in vivo* were taken using the Living Imaging System (IVIS Lumina XRNS Ⅲ; Perkin Elmer, Germany). The organs including brain, lung, heart, spleen, liver, and kidney were also harvested at the corresponding time-points and labeled-EVs were quantitated *ex situ* using the same imaging system. After that, the organs were cut into frozen tissue slides. Slides were stained with DAPI and observed under a fluorescence microscope for labeled DiR-EVs.

To detect the uptake of BHD-NSC-EVs by NSCs, BHD-NSC-EVs were first labeled with 10^−6^ m PKH26 Cell Membrane Labeling Dye (Sigma-Aldrich, United States) following the manufacturer’s protocol. After quenched by PBS containing 10% bovine serum albumin (BSA), excess dye was removed by centrifugation at ×10,000 g for 30 min at 4°C; the PKH26 labeled EVs were harvested by ultracentrifugation at 100,000 g for 1 h at 4°C. Labeled BHD-NSC-EVs were added and co-cultured with NSCs for 2 h. Then, the cells were washed twice with PBS and fixed with PFA (4%, w/v) for 30 min at 4°C. The fixed cells were washed with PBS and placed on the glass slide. After performing the immunofluorescent staining procedures with NSC marker Nestin antibody (1:200, Abcam, United States), The uptake of PKH26-BHD-NSC-EVs was then observed under a confocal microscope (LSM880, Zeiss, Germany).

### 2.14 Preparation of tissue sections

After 28 days of MCAO and treatment later, rats were anesthetized with a lethal dose of isoflurane (3% maintenance). The thoracic cavity was opened, an empty needle was inserted into the heart and a small gap in the right atrial appendage was cut. Cold saline was infused through the heart until the viscera of the rat became colorless and liquid overflowed from the right atrial appendage, indicating that the blood had been replaced by saline. PFA (4% w/v) was then infused until the limbs and trunk of the rat became stiff. The brain and other organs were then removed and was fixed in PFA at 4°C overnight, followed by 30% sucrose/PBS until the organs had sunk to the bottom of the container. Organs were then cut into 20 and 8 μm thick tissue sections to detect infarct volume and IF respectively through a freezing microtome (Thermo Fisher Scientific, United States). Sections were then collected onto a poly-Dlysine-coated anti-offset slide. All slides were stored at −80°C for further analysis.

### 2.15 IF staining of cultured cells and tissue sections

Cells and tissue sections treated as described in 2.3., and finally incubated overnight at 4°C with the following primary antibodies: anti-Nestin (1:500, mouse IgG1; BD Biosciences, United States), anti-SOX2 (1:500, rabbit IgG; Abcam, United States), anti-MAP2 (1:500, rabbit IgG; Abcam, United States), anti-NeuN (1:800, mouse IgG; Abcam, United States), anti-GFAP (1:1,000, rabbit IgG; Cell Signal Technology, United States). Anti-BrdU (1:500, mouse IgG1; BD Biosciences, United States), anti-vWF (von Willebrand factor, 1:200, rabbit IgG; Abcam, United States), anti-PSD95 (1:500, mouse IgG; Abcam, United States). The following day, the cells and tissue sections were treated with goat anti-mouse IgG H&L (1:2000, Alexa fluor 647), goat anti-rabbit IgG H&L (1:2000, Alexa fluor 488) at room temperature for 2 h and the nuclei were counterstained for 10 min with DAPI. Immunoreactivity was visualized using a fluorescence microscope (AXIO Vert. A1&Imager A2, Carl Zeiss Microscopy GmbH, Germany). ImageJ software (NIH, Bethesda, MD) was used to randomly select five fields under the microscope and compare five views, The number of staining positive cells in the field was calculated by taking the mean of five fields to calculate the cell density. At the same time, the average fluorescence intensity of PSD95 in brain tissue was measured by Image J software (NIH, Bethesda, MD).

### 2.16 WB analysis

Total protein was extracted from cells and EVs, and the protein concentration was determined by BCA assay as instruction described. The proteins samples were electrophoresed by SDS-PAGE (Sodium Dodecyl Sulfate Polyacrylamide Gel Electrophoresis) with 20 μg on the lane and then transferred onto polyvinylidene difluoride membranes for 1 h. Membranes were blocked with 5% BSA for 2 h at room temperature and incubated with following antibodies overnight at 4°C: CD9 (1:1,000, rabbit IgG; Cell Signal Technology, Danvers, MA, United States), CD81 (1:1,000, rabbit IgG; Cell Signal Technology), Calnexin (1:1,000, rabbit IgG; Abcam, United States), ALIX (1:1,000, rabbit IgG; Abcam, United States), TSG101 (1:1,000, rabbit IgG; Abcam, United States), Synapsin (1:1,000, mouse IgG1; Abcam, United States), PSD-95 (1:1,000, mouse IgG1; Abcam, United States), *β*-Actin (as a gel-loading control, 1:1,000, rabbit IgG; Abcam, United States). After rinsed with 1 × TBST solution, 10 min × 3 times; the membranes were then incubated with HRP conjugated sheep anti-rabbit IgG or anti-mouse IgG (1:2000, Thermo Fisher Scientific, United States) for 2 h at room temperature; following rinsed with 1 × TBST, 10 min × 3 times; ECL chemiluminescence was performed to visualize the immunolabeled bands using an enhanced chemiluminescence reagent (Thermo Fisher Scientific, United States). Image J software (NIH, Bethesda, MD) was used to analyze the mean optical density of protein expression bands.

### 2.17 qRT-PCR

Total RNA from cells and EVs were extracted using Trizol (Tiangen, Beijing, China). The RNA is then reversely transcribed into cDNA, reaction conditions: 37°C for 60 min, 85°C for 5 s. Using cDNA as template, 2 × PCR Mix, upstream and downstream primers and sterilized double steaming water were added to a total volume of 20 μL, mixed, and put into the PCR instrument. The PCR amplification procedure was as follows: predenaturation at 95°C for 10 min; Denaturation at 95°C for 10 s, annealing at 60°C for 20 s, extension at 72°C for 15 s, 45 cycles, extension at 72°C for 10 min, cooling at 25°C for 30 s. Three wells were set for each sample, *β*-Actin and U6 was used as the internal reference primers. The primers used were synthesized by Sangon Biotech Company (Shanghai, China) in this study. The relative miRNA expression of target genes was calculated by the 2^−△△Ct^ method. Following was the primer sequence for qRT-PCR involved in this study:

**Table:** 

miRNA/mRNA	Forward (5′-3′)	Reverse (5′-3′)
rno-mir-124-5p	CGT​GTT​CAC​AGC​GGA​CCT​TGA​T	TGGTGTCGTGGAGTCG
rno-mir-9a-5p	CGC​TCT​TTG​GTT​ATC​TAG​CTG​TAT​GA	TGGTGTCGTGGAGTCG
rno-mir-137-5p	CGA​CGG​GTA​TTC​TTG​GGT​GGA​TAA	TGGTGTCGTGGAGTCG
rno-mir-184	TGG​ACG​GAG​AAC​TGA​TAA​GGG​T	TGGTGTCGTGGAGTCG
U6	CTCGCTTCGGCAGCACA	AAC​GCT​TCA​CGA​ATT​TGC​GT
β-actin	CGT​CCG​TGA​CAT​CAA​GGA​GAA​GC	ACC​GAG​GAA​GGA​AGG​CTG​GAA​G

### 2.18 CCK-8 detection for cell proliferation

The treated neurospheres of each group were made into single cell suspension of NSCs and planted in a 96-well plate at a density of 2 × 10^4^/well. Then the cells were treated with EVs from different groups or MP-BHD or PBS. The NSCs were co-cultured with 15 μg/mL EVs in culture medium, which was set as medium-dose group (NSC-EVs-M or BHD-NSC-EVs-M), the low-dose group (NSC-EVs-L or BHD-NSC-EVs-L) and high-dose group (NSC-EVs-H or BHD-NSC-EVs-H) with 7.5 and 30 μg/mL EVs respectively. The final concentration of MP-BHD in medium is 10% of the reaction system which was set as MP-BHD group, the PBS group contains the same volume of sterile PBS. The culture medium was added in a circle around the 96-well plate and 100 μL medium of the corresponding group was added to each well according to the experimental sequence for 48 h, following 10 μL CCK-8 solution with 10% concentration, which was added to each well and incubated at 37°C for 6 h. The OD value was measured at 450 nm with a full-wavelength microplate reader. Each group of cells was set up with three re-wells, and each experiment was repeated three times. The OD value is directly proportional to the number of viable cells in the culture system.

### 2.19 Differentiation of NSCs

The differentiation of NSCs was carried out as previously described (Yuan et al., 2021). The treated neurospheres of each group were made into single cell suspension of NSCs and planted on Matrigel-coated coverslips in a 24-well plate at a density of 2 × 10^5^/well. Then the cells were treated with EVs or MP-BHD or PBS. The NSCs were co-cultured with 15 μg/mL NSC-EVs or BHD-NSC-EVs in culture medium. The final concentration of MP-BHD in medium is 10% of the reaction system was set as MP-BHD group, The PBS group contains the same volume of sterile PBS. The medium was changed every 3 days. After 1 week, removed the medium, cells were fixed with paraformaldehyde (PFA, 4%, w/v, pH 7.4), permeated with 0.3% Triton X-100, and blocked with goat serum. The cells were labeled with the neurons markers Map2 antibody and glial cells markers GFAP antibody at 4°C overnight, and then treated with fluorescent secondary antibody at room temperature for 2 h. The nuclei were stained with 4′, 6-diamidino-2-phenylindole (DAPI), and the plates were sealed and fixed, and detected by immunofluorescence (IF) staining with fluorescence microscopy. Then observed and counted the MAP2^+^/DAPI and GFAP^+^/DAPI respectively, the total cell number was counted by the number of DAPI in the same size field of view. The proportion of positive cells was calculated.

### 2.20 Flow cytometry analysis for cell cycle

The treated neurospheres of each group were made into single cell suspension of NSCs .5 × 10^5^ cells were collected, centrifuged, and the supernatant was discarded. The cells were washed twice with precooled PBS, and the precooled volume fraction was 70% ethanol. The cells were fixed at 4°C overnight. After centrifugation, the cells were collected, washed once with PBS, then 500 μL PBS (containing 50 mg/L propidium bromide, 100 mg/L RNase A, 0.2% Triton X-100) was added, and the cells were incubated at 4°C for 30 min in the dark room. Standard procedures were used for flow cytometry detection. A total of 2 × 10^5^ or 3 × 10^5^ cells were counted. The results were analyzed by cell cycle fitting and ModFit software.

### 2.21 Transwell migration assay

Boyden chamber assays were performed using 24-well transwell inserts (Corning, NY, United States) with 8 μm poresized filters and 24-well culture plates as described previously. NSCs (5 × 10^4^ cells per well; 3 replicates per group) were suspended in DMEM/F12 medium and plated into the upper chamber. 50050μL DMEM/F12 medium with PBS, MP-BHD (50 μL), NSC-EVs and BHD-NSC-EVs (100 μg/mL) was added to the lower chamber. After incubation for 24 h, NSCs attached to the upper surface of the filter membranes were removed by cotton swabs and cells on the bottom side of the filter (the migrated cells) were stained with 0.5% crystal violet for several minutes. The number of migrated cells was quantified under an optical microscope at a ×100 magnification (Leica, Tokyo, Japan). The OD value of each well was measured at 550 nm by a microplate reader (Bio-Rad 680, Hercules, United States) and cell migration was represented through the mean OD value of each individual well.

### 2.22 Next-generation sequencing of EVs

Total RNA extracted from the NSC-EVs and BHD-NSC-EVs was used for miRNA arrays. miRNA profiles were performed with OE Biotech’s (Shanghai, China) miRNA microarray service based on Affymetrix miRNA 3.0 Array. The raw fasted data quality was checked by the FASTQC, meanwhile, the adaptor was removed to trim quality bases by the Trimmomatic. The leading and trailing ambiguous or low-quality bases, which were below Phred quality scores of 3, were also removed after adapter clipping. The miRNA read counting was detected by the Chimirra and the miRNA expressions were normalized by the trimmed mean of M-values (TMM). The edge program was further used to identify the differentially expressed genes. The gene with a fold change of expression more than 2 was defined as a differentially expressed one. The miRNA target gene prediction was detected through TargetScan (http://targetscan.org/) and miRDB (http://www.mirdb.org/). And the cluster Profiler R was also performed to conduct the Gene Ontology (GO) (http://www.geneontology.org/) and Kyoto Encyclopedia of Genes and Genomes pathway (KEGG) (http://www.genome.jp/kegg/) enrichment analyses.

### 2.23 Statistical analysis

GraphPad Prism 9.0 (GraphPad Software, Inc., La Jolla, CA, United States) was run to process and analysis the data and images. All values are expressed as mean ± s.e.m. A two-tailed Student’s *t*-test or one-way ANOVA with *post hoc* tests, as appropriate. *p* < 0.05 was considered as statistically significant differences for all tests.

## 3 Result

### 3.1 Identification of the characteristics of the obtained NSCs and EVs

**FIGURE 1:**
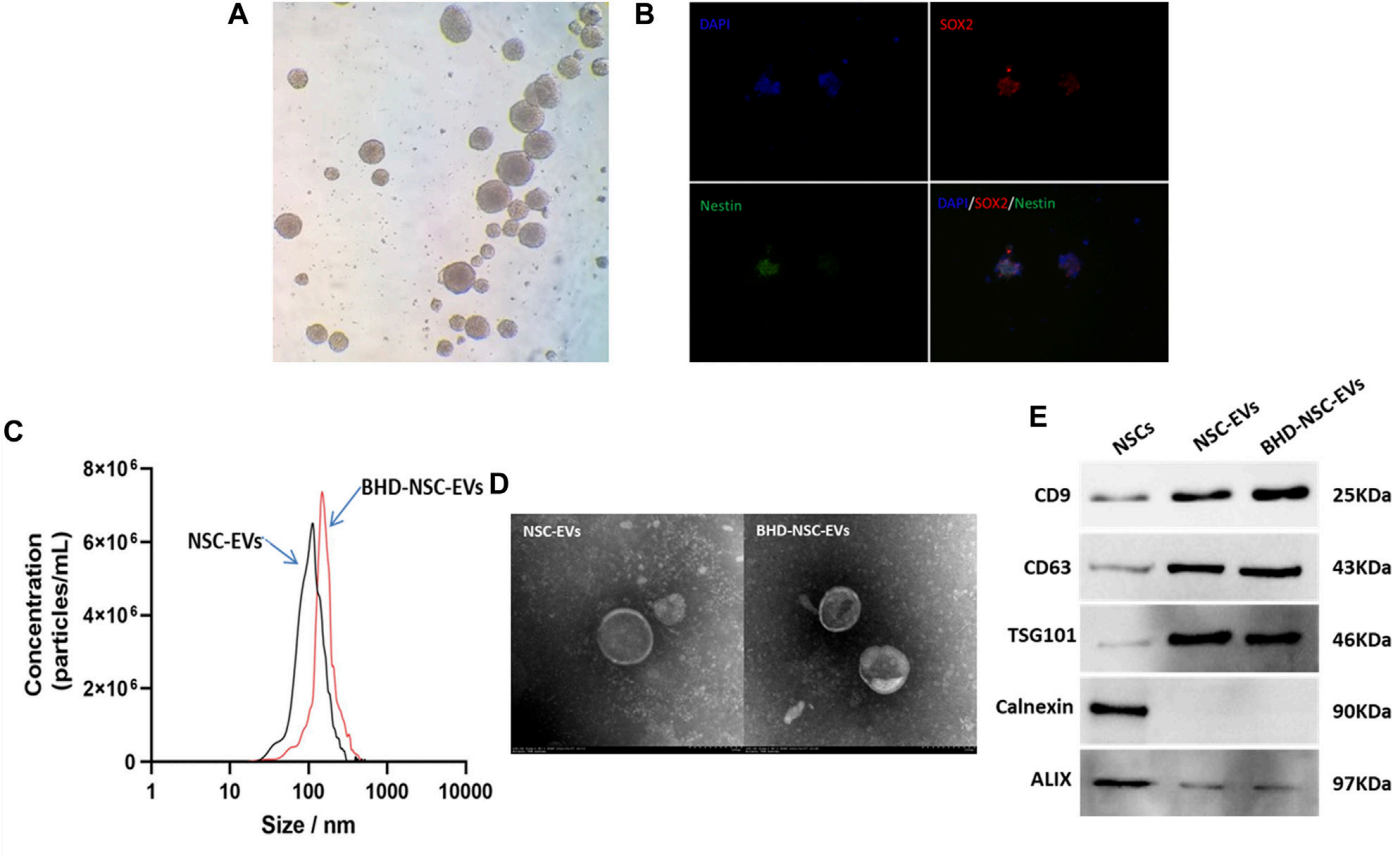
Identification of primary neural stem cell and extracellular vesicles. **(A)** NSCs were visualized and grew in sphere-like cell clusters under ordinary optics microscope. **(B)** Immunofluorescence staining of adherent neurospheres showed expression of the NSC markers nestin (red) and SOX2 (green) under fluorescence microscope. **(C)** Particle size distribution were analyzed by nanoparticle tracking analysis (NTA), which indicated NSC-EVs and BHD-NSC-EVs were different particle size slightly. **(D)** Transmission electron microscopy (TEM) images revealed small lipid bilayer membrane vesicles morphology of BHD-NSC-EVs scale bar: 100 nm. **(E)**Western blot analysis was conducted to detect the expression of specific extracellular vesicle-related positive markers (CD63, CD9, TSG101) and negative markers (Calnexin, ALIX) in BHD-NSC-EVs and NSCs. NSC-EVs, Neural Stem Cells Derived Extracellular Vesicles; BHD-NSC-EVs, Medicated Plasma of Buyang Huanwu Decoction-Preconditioned Neural Stem Cells Derived Extracellular Vesicles.

To obtain NSC derived EVs, primary NSCs were extracted from fetal rat brains following previously described methods, with some modification. Briefly, under sterile conditions, the cerebral cortexes of embryonic day 13.5 (E13.5) rat brains tissues were dissected and dissociated in PBS. Following centrifugation at a low speed, the tissues suspension was collected and filtered through a 40-μm filter to form free single cells. These cells were then cultured in a growth medium at 37°C and 5% CO_2_. Using an ordinary optics microscope, the NSCs were visualized, and were observed to grow in sphere-like cell clusters (Figure 1A), which were found to be immune-positive for the stem cell markers SOX2 (red fluorescence) and nestin (green fluorescence), under fluorescence microscope (Figure 1B). EVs were isolated from the culture supernatant of the untreated NSCs and the NSCs treated with MP-BHD and PBS using a combination of centrifugation, ultrafiltration, and ultracentrifugation. NTA was performed to identify the size and number of EVs, which revealed a particle size distribution of between 50 and 150 nm in the NSC-EVs and between 80 and 180 nm in the BHD-NSC-EVs (Figure 1C). TEM analysis revealed typical EV structures of membrane-bound nanovesicles (Figure 1D). In addition, WB was performed to detect the EV surface markers, CD63, CD9, and TSG101, which had a high expression on the surface of both the NSC-EVs and the BHD-NSC-EVs, while calnexin and ALIX were not detected (Figure 1E). Taken together, these analyses confirmed that NSCs and NSC-EVs were successfully obtained.

### 3.2 Effect of MP-BHD on the secretion of EVs by NSCs

**FIGURE 2:**
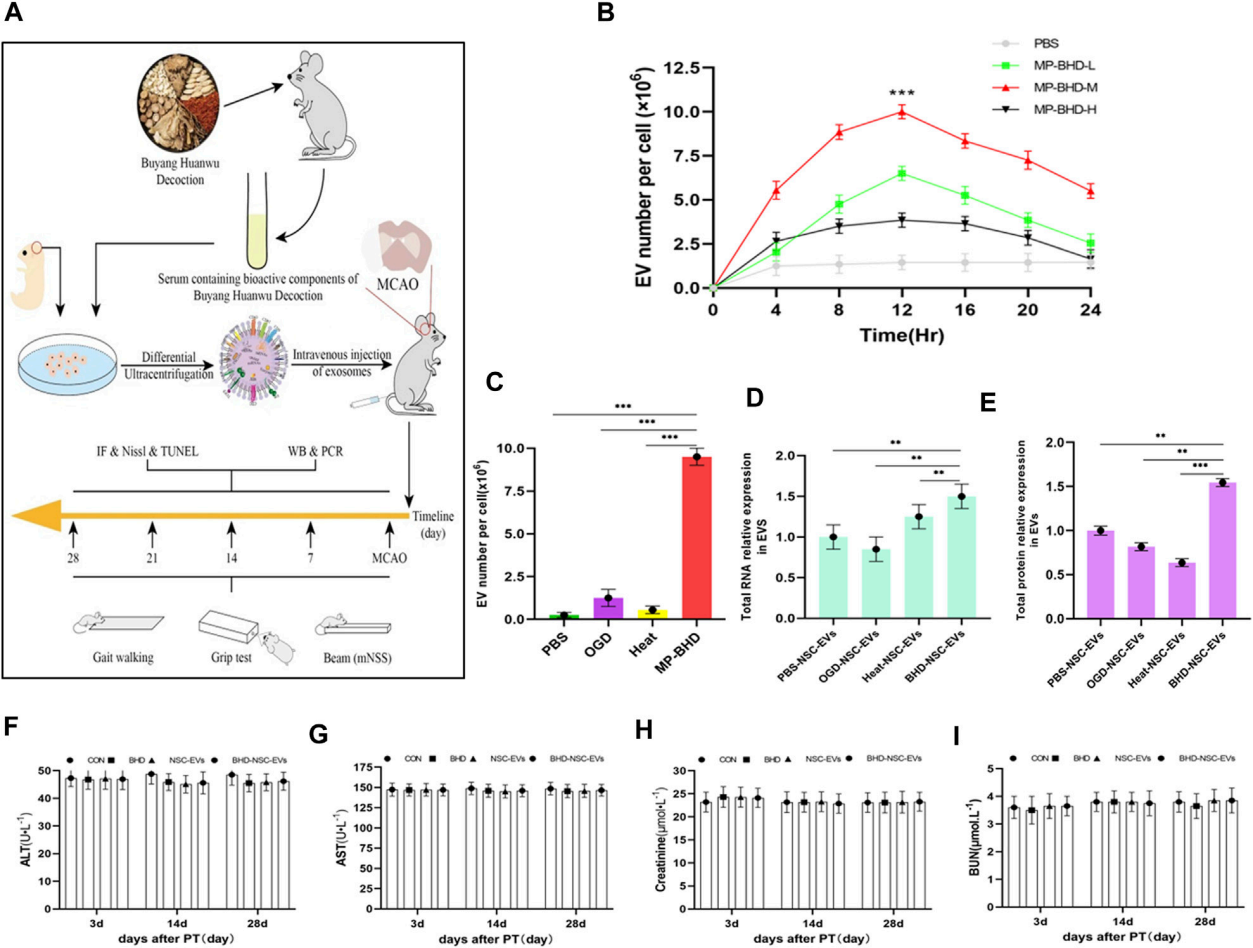
*In vitro*, NSCs preconditioned by MP-BHD generated large quantities of EVs loading higher content. **(A)** Schematic representation of the examination, harvest and administration of MP-BHD and BHD-NSC-EVs, the therapeutic effect of BHD-NSC-EVs *in vivo* through MCAO model rats **(B)**EVs secretion from NSCs peaks around 12 h after being preconditioned with MP-BHD, the number of EVs increase when the dosage was increased from low (5% of the reaction system) to medium dosage (10% of the reaction system), but high dosage (20% of the reaction system) MP-BHD generated low yield of EVs conversely. (*n* = 3), ***p < 0.001, MP-BHD –M vs. PBS. **(C)** MP-BHD significantly increased the number of EVs yield compared with traditional methods of stress-induced EV release, including OGD and heat treatment. MP-BHD, NSCs were cultured in DMEM treated with conventional dosage (10% of the reaction system) MP-BHD for 12 h in cell incubator with 5% CO_2_ at 37°C. OGD, NSCs were cultured in a buffered salt solution without glucose in an anaerobic chamber at 37°C humidified environment for 8 h and then transferred to normal cell culture conditions. Heat stress, NSCs were cultured at 42°C for 2 h and then transferred to 37°C with normal cell culture conditions. (*n* = 3), ***p < 0.001. **(D–E)** Contents amount encapsulated in EVs produced by an equal number of NSCs stimulated with different methods of stress-induced EV release was measured by nanodrop (**(D)** RNA expression levels) and BCA assay (**(E)** protein expression levels). BHD-NSC-EVs contain higher RNA and protein content than other EVs. (*n* = 3), **p < 0.01, ***p < 0.001. **(F–I)** The adverse reactions of BHD and BHD-NSC-EVs were determined according to serum hepatic enzymes and renal filtration function. ALT **(F)** and AST **(G)** presented no hepatotoxicity, BuN **(H)** and Cr **(I)** presented no nephrotoxicity. All data are from three independent experiments and are presented as mean ± s.e.m, two-sided Student’s *t*-tests were used for comparisons **(B–E)**. NSCs, Neural Stem Cells; MP-BHD, Medicated plasma of Buyang Huanwu Decoction; EVs, Extracellular vesicles; BHD-NSC-EVs, Medicated Plasma of Buyang Huanwu Decoction-Preconditioned Neural Stem Cells Derived Extracellular Vesicles; MCAO, middle cerebral artery occlusion. OGD, Oxygen glucose deprivation; BCA, bicinchonininc acid. ALT, alanine aminotransferase; AST, aspertate aminotransferase; BuN, blood urea nitrogen; Cr, creatinine; NS, not significant.

To explore whether BHD affects the secretion of EVs from NSCs, primary NSCs from fetal rats were cultured and preconditioned under different conditions. BHD is a complex, poly-juice potion, containing a variety of natural wild herbs. To approximate the real utilization situation *in vivo*, MP-BHD was prepared. In brief, healthy rats were given intragastric administration of BHD for 3–7 days, with blood then collected through the abdominal aortic and allowed to clot before being centrifuged and filtered (BHD group). Using the same method, healthy rats were treated with PBS and allocated to the control group (PBS group) (Figure 2A). An increase in the yield of EVs released as a dosage of MP from low to medium, was observed, but a high dosage resulted in a low yield within 12 h. The EVs release yield continued to increase prominently over a 24 h period, reaching peaked at 12 h following MP-BHD stimulation (Figure 2B). In term of the effect of PBS, OGD and heat stimulation, the NSCs were stimulated to produce and release extremely small amounts of EVs. In contrast, MP-BHD stimulation significantly promoted the NSCs to secrete a large number of EVs (BHD-NSC-EVs) (Figure 2C). 6 × 10^6^ NSCs were cultured in 10 mL NSC proliferation medium for 12 h, NTA analyzed the concentration of NSC-EVs was 8 ± 0.5 × 10^11^ particles/mL and BHD-NSC-EVs was 6.5 ± 0.5 × 10^12^ particles/mL (under a medium dosage); BCA protein assay kit determined the protein concentration was 1.44 ± 0.06 μg/μL and 2.15 ± 0.05 μg/μL respectively. The BHD-NSC-EVs were compared with others derived-EVs through the total RNA and protein concentration. Hence, both the RNA and the protein concentrations in the BHD-NSC-EVs were significantly higher than in the other solutions, largely due to the complicated make up of BHD (Figures 2D, E). In consideration of the complex components of the Chinese herbs and their formulations, of which the most common adverse reactions include liver and renal toxicity, were determined according to serum hepatic enzymes and renal filtration function, including aspartate aminotransferase (AST), alanine aminotransferase (ALT), blood urea nitrogen (BuN), and creatinine. The test results revealed that there was no difference in concentration compared to healthy rats, and that BHD and BHD-NSC-EVs are non-toxic (Figures 2F–I). These findings indicate that the MP-BHD promoted the large-scale generation of NSCs derived EVs, which contain a large amount of protein and RNA and are non-toxic. As such, these BHD-NSC-EVs may have special pharmacological effects.

### 3.3 BHD-NSC-EVs can enrich in brains and be taken up by NSCs

**FIGURE 3:**
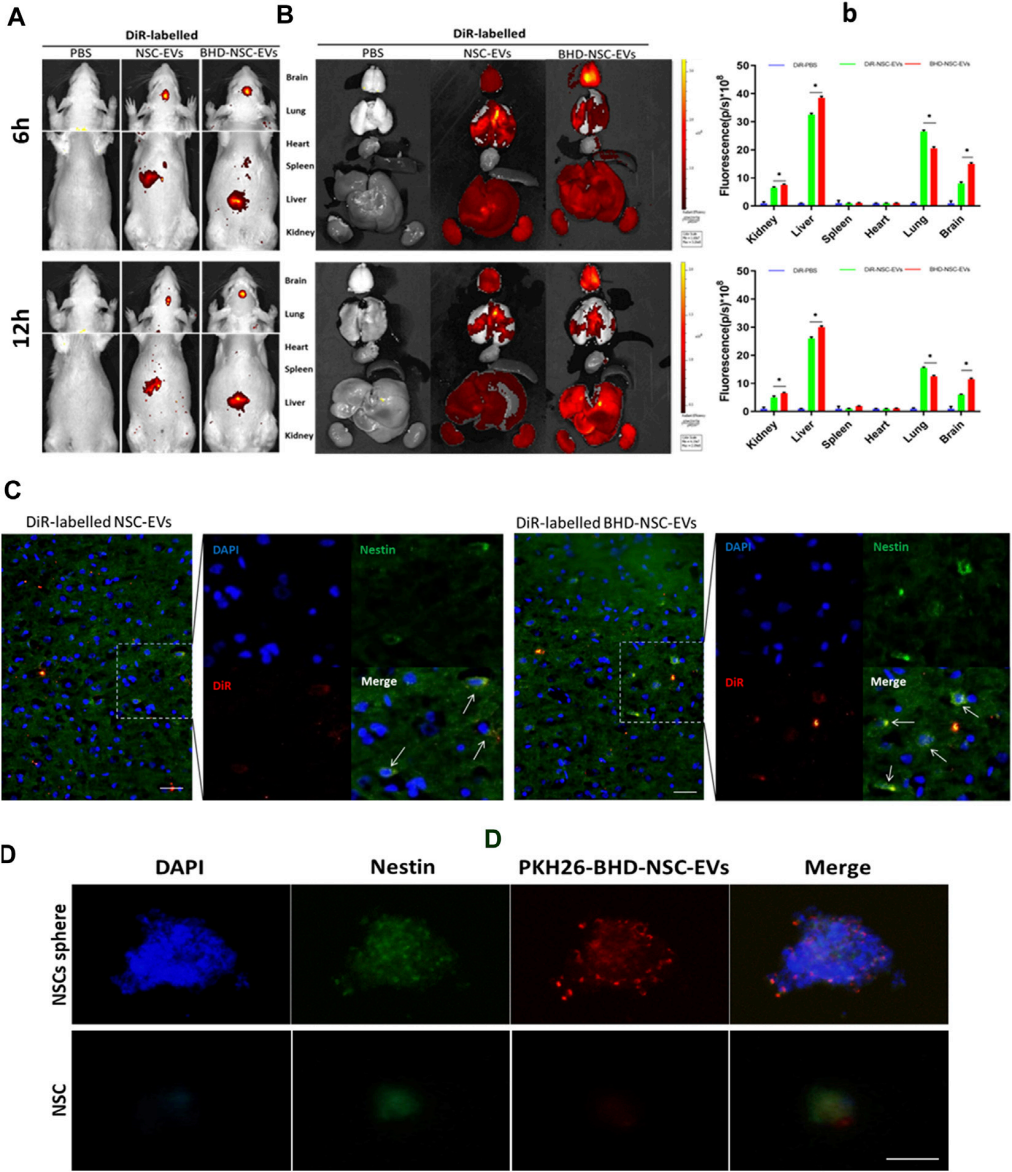
The biodistribution of BHD-NSC-EVs *in vivo* and *in vitro*. **(A)** Representative IVIS images showed the DiR-labeled NSC-EVs and BHD-NSC-EVs were intravenously injected through caudal vein after 6 and 12 h and mainly accumulated in the heads of MCAO model rats using the Bruker Small Animal Optical Imaging System. **(B-b)** Representative IVIS images of six different organs including the brain, lung, heart, spleen, liver, and kidney harvested after 6 and 12 h following caudal vein injection of DiR-labeled NSC-EVs and DiR-labeled BHD-NSC-EVs, respectively. Quantification of the fluorescent intensity expressed as the means ± SEMs (*n* = 17/time point) demonstrated the biodistribution of BHD-NSC-EVs and NSC-EVs in six different organs. (*n* = 5); ns, no significance; **p < 0.01, BHD-NSC-EVs vs. NSC-EVs **(C)** Representative immunofluorescent images of brain sections revealed the DiR-labeled BHD-NSC-EVs and NSC-EVs (red) could be taken up by endogenous NSCs (green/blue). **(D)** The uptake of PKH26-labeled BHD-NSC-EVs (red) in nestin^+^ NSCs sphere or NSC (green) was visualized using confocal microscopy, the scale bar: 20 µm. All data are from three independent experiments and are presented as mean ± s.e.m, two-sided Student’s *t*-tests were used for comparisons **(B)**. IVIS, infrared video data imaging system; NSC-EVs, Neural Stem Cells Derived Extracellular Vesicles; BHD-NSC-EVs, Medicated Plasma of Buyang Huanwu Decoction-Preconditioned Neural Stem Cells Derived Extracellular Vesicles.

To verify whether BHD-NSC-EVs can act on stroke lesions, the biodistribution of transfused BHD-NSC-EVs *in vivo* was determined using the MCAO model rats. 50 rats were involved in MCAO operation. 24 h after surgery, nine rats died and seven rats with scores of 0 and 4 were withdrawn from the experiment. Following the experiment, the rats (with scores 1–3) were randomly divided into 2 batchs (6 and 12 h), three groups/batch. Groups: PBS group *n* = 3; NSC-EVs group *n* = 7; BHD-NSC-EVs group *n* = 7. In this process, DiR was utilized to label the BHD-NSC-EVs, the NSC-EVs, and the supernatant (EV free). The EVs labeled using DiR were administered *via* tail-intravenous injection following stroke onset. 6 and 12 h following the injection, The MCAO model rats underwent *in vivo* fluorescent imaging using IVIS (Figures 3A-b), with the results indicating that the DiR fluorescence was mainly localized in the head and liver area of MCAO rats (Figure 3A), following this, the rats were sacrificed and the fresh organs including the heart, liver, spleen, lung, and kidneys were harvested and imaged *in vitro* for the subsequent experiments. EVs were mostly detected in the liver and lung and then in the kidneys. In the brain, there were plenty of EVs detected, which affirmed that the BHD-NSC-EVs could enriched in the brain of MCAO rats (Figures 3B-b). Furthermore, whether the EVs could be taken up by NSCs or not was investigated. The experiments of endogenous NSCs uptaking NSC-EVs and BHD-NSC-EVs were conducted through immunofluorescent analysis of brain tissue sections, the results shown the DiR–labelled NSC-EVs and BHD-NSC-EVs could be taken in by endogenous NSCs (Figure 3C **)**. At the same time, BHD-NSC-EVs were labeled PKH26 and incubated with nestin-positive NSCs sphere or NSC. The confocal images revealed colocalization of PKH26-labelled BHD-NSC-EVs (red) with the NSC markers nestin (green), suggesting that NSCs sphere or NSC could take up BHD-NSC-EVs (Figure 3D). Overall, these findings suggest that BHD-NSC-EVs can target the brain and enter NSCs to educes the biological effects.

### 3.4 Acceleration of neurological function recovery of MCAO models by BHD-NSC-EVs

**FIGURE 4:**
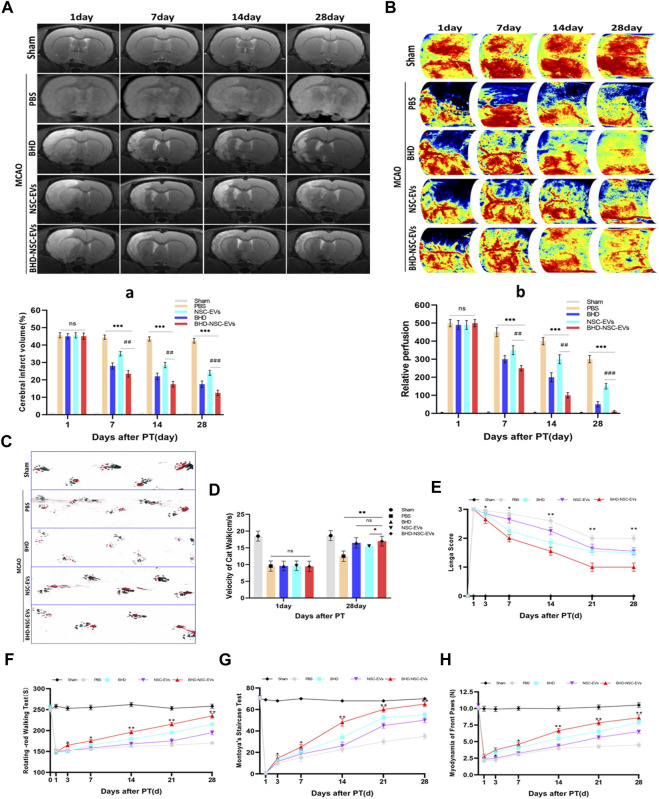
The therapeutic effect of BHD-NSC-EVs on the acquired neurological impairment after ischemic stroke. **(A-a)** T2-weighted images (T2WI) of MRI revealed the changed infarct volume of brain ischemia rats after MCAO and treatment at different test period, the ischemic lesion shown in white using ImageJ in BHD-NSC-EVs group reduced more obvious than that in other groups. (*n* = 7/group), ns: no significance. ^##^ *p* < 0.01, ^###^ *p* < 0.001, ***p < 0.001. **(B-b)** Representative images of Laser speckle of cerebral blood flow showed the changed cerebral vascular perfusion after MCAO and treatment at different test period. BHD-NSC-EVs significantly promoted cerebral vascular perfusion recovery relative to other treatments. *n* = 7 rats/group, ns: no significant. (*n* = 7/group), ns: no significance. ^##^ *p* < 0.01, ^###^ *p* < 0.001, ***p < 0.001. **(C,D)** Gait analysis after MCAO and treatment or not. **(C)**Representative footprints of rats walking 28 days, blue and red represent front paw and hindpaw prints, respectively, drag marks indicated lack of walking coordination. **(D)** velocity of cat walk test revealed significantly faster gait recovery after treatment of BHD-NSC-EVs than others. (*n* = 7/group), ns: no significant. *p < 0.05, **p < 0.01.**(E)** Longa score was assessed to evaluate neurological deficits after MCAO and treatment or not. (*n* = 7) *p < 0.05, **p < 0.01 BHD-NSC-EVs vs. PBS **(F)** Rotating-rod walking test evaluated fatigue resistance of motor function after MCAO and treatment or not. (*n* = 7/group), *p < 0.05, **p < 0.01 BHD-NSC-EVs group vs. PBS group **(G,H)** Front paws myodynamia of rats after MCAO and treatment or not were detected by montoya’s staircase test and myodynamia of frontpaws, BHD-NSC-EVs markedly strengthened the myodynamia of front paws after MCAO relative to other treatments. (*n* = 7/group), *p < 0.05, **p < 0.01 BHD-NSC-EVs group vs. PBS group. All data are from three independent experiments and are presented as mean ± s.e.m, two-sided Student’s *t*-tests were used for comparisons ,**(a,b,D-H)**.

To evaluate the therapeutic effects of BHD-NSC-EVs on the neurological impairment induced by an ischemic stroke, models using Sprague-Dawley rats were built with MCAO as previously described (Gao et al., 2020). 60 rats were involved in operation, in which 5 rats in sham operation and 55 rats in MCAO operation. Twenty four hours after surgery, 12 rats died, which were all from MCAO operation. 13 rats with scores of 0 and 4 were withdrawn from the experiment. 2 rats were used for immediate TTC staining to evaluate the effect of MCAO surgery. Following the operation, the rats (with scores 1–3) were randomly divided into five groups (Supplementary Figure S1): the Sham group (skin incised but not MCAO, treated with nothing, *n* = 5); the PBS group (intravenous injection of PBS, *n* = 7); the BHD group (intragastric administration of BHD, *n* = 7); the NSC-EVs group (intravenous injection of NSC-EVs, *n* = 7); and BHD- NSC-EVs group (intravenous injection of BHD- NSC-EVs, *n* = 7). Using different time points, the rats underwent MRI, LSIS of cerebral blood flow, and motor function assessments. The MRI results revealed a similar size of cerebral infarctions 1 day following operation. As the treatment progressed, the lesion size was gradually reduced, with the lesion size in the BHD- NSC-EVs group decreasing more significantly than in the other groups at the same time point (Figures 4A-a). The LSIS of cerebral blood flow revealed the occurrence of severe cerebral ischemia on the infarct-side, with the lesion side undergoing hypoperfusion following the operation. As the treatment progressed, the cerebral blood perfusion gradually recovered, which was more obvious in the BHD-NSC-EVs group at the same time point (Figures 4B-b). Following MCAO, the coordination of forepaw−hindpaw movements and the velocity of catwalk decreased immediately, as determined *via* gait analysis, while as the treatment progressed, the BHD-NSC-EVs group exhibited improved motor coordination and significantly faster gait recovery than the other groups (Figures 4C, D).

Animal behavior tests were performed to assess the motor function of the MCAO rats in term of Longa score, coordination of the forepaw−hind paw movements, a fatigue resistance test, and a muscle strength test from day 1 to day 28 following stroke onset and treatment. Compared with the PBS group, from day 7 to day 28, the BHD group, BHD-NSC-EVs group, and NSC-EVs group exhibited neurological functional recovery improvement (Longa scores), with the BHD-NSC-EVs group demonstrating the fastest recovery (Figure 4E). The fatigue resistance test of motor functioning was conducted using rotating-rod walking test, and the MCAO rats exhibited significant long-term motor function deficits, which were most prominent from day 1 to day 3 following MCAO. As the treatment progressed, the sustained exercise capacity increased from day 7 to day 28, with the BHD- NSC-EVs group demonstrating significantly more durable exercise persistence capacity than the other groups (Figure 4F). The front paws myodynamia of the rats was assessed using Montoya’s staircase test. Hence, the BHD, BHD-NSC-EVs, and NSC-EVs strengthened the myodynamia and enabled the MCAO rats to grab effectively, with the BHD-NSC-EVs group exhibiting greater improvement than the other groups at the same time points (day 7 to day 28). During the test period, the Sham and PBS group remained unchanged compared with the other groups (Figures 3G, H). Collectively, these results demonstrated the significant effect of the BHD-NSC-EVs on the long-term recovery of the motor function of the MCAO rat models, and indicated that BHD-NSC-EVs could effectively accelerate neurological function recovery following stroke.

### 3.5 BHD-NSC-EVs promoted the neurovascular unit remodeling after stroke

**FIGURE 5:**
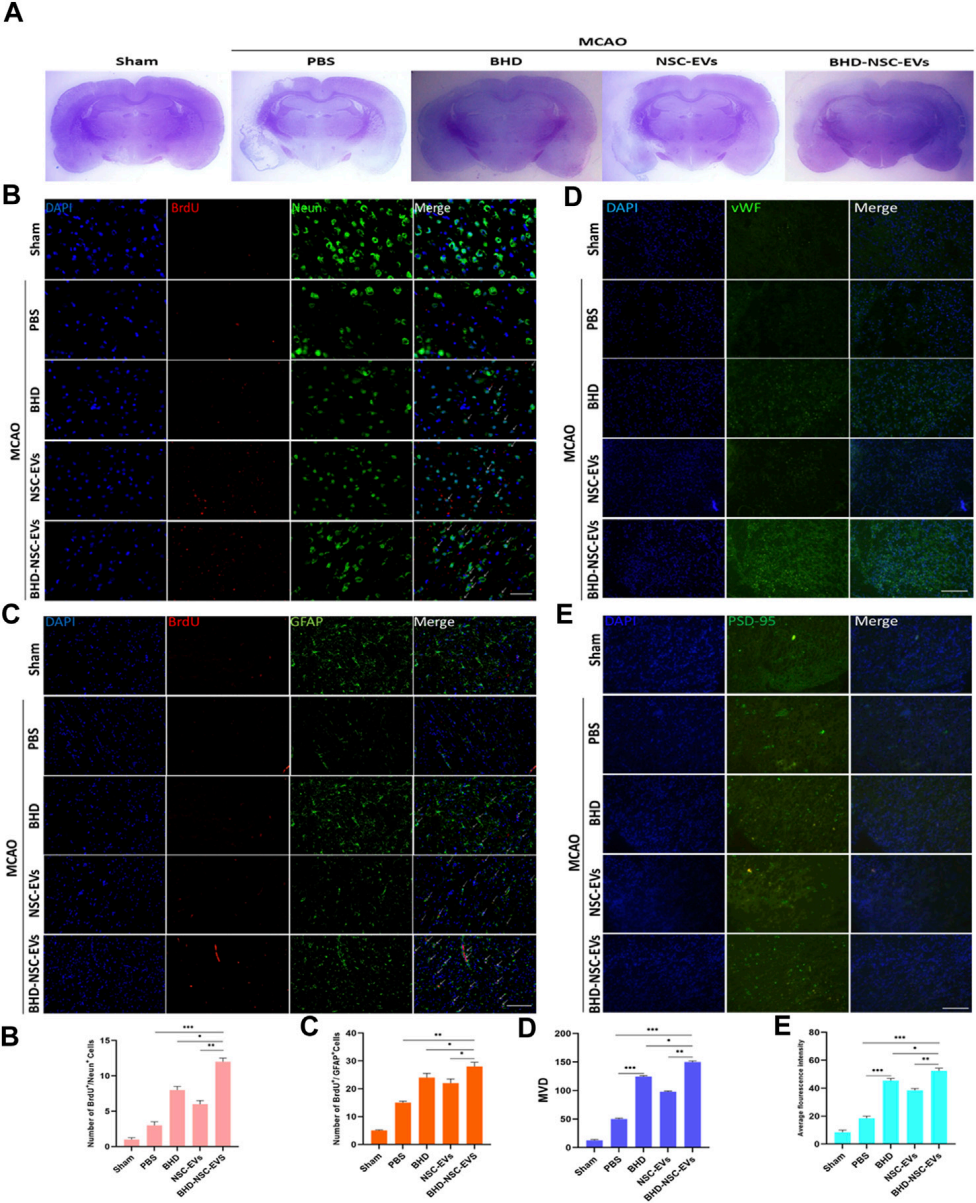
BHD-NSC-EVs promoted the neurovascular unit remodeling after stroke. **(A)** Representative nissl-stained coronal section of cerebral infarcted lesion in MCAO rats. (*n* = 7/group) **(B-c)** Immunofluorescent analysis of neurogenesis of neuros and glial cells, fluorescent staining with DAPI (blue), BrdU (red), Neun (green) and GFAP (green) revealed neurogenesis of **(B-b)** neuros (BrdU+/Neun+) and **(B-b)** glial cells (BrdU+/GFAP+). (n = 7/group), *p < 0.05, **p < 0.01, Scale bar 100 μm in panel **(D)** Immunofluorescent analysis of neurogenesis of angiogenesis with vWF (green). (*n* = 7/group), *p < 0.05, **p < 0.01, ***p < 0.001, Scale bar 100 μm in panel **(E)** Immunofluorescent analysis of a marker of synaptic plasticity with PSD-95 (green). **(a-d)** Quantitative data of number of regenerative cells and fluorescence intensity of PSD-95 using ImageJ. (*n* = 5). NS, not significant. *p < 0.05, **p < 0.01, ***p < 0.001. All data are from three independent experiments and are presented as mean ± s.e.m,two-sided Student’s t-tests were used for comparisons **(b-e)**.

To provide further evidence on the role that BHD-NSC-EVs play in the treatment of neurological function recovery, pathological examination was performed. Nissl staining revealed the lesion area of MCAO changed in the treatment group 4 weeks post MCAO onset, a significant residual loss of MCAO tissue in the PBS group was observed while the lesion area was significantly smaller in the BHD-NSC-EVs than others (Figure 5A; Supplementary Figure S5A). We mainly detected the neurovascular unit regeneration through immunofluorescent analysis of neurogenesis of neuros and glial cells and angiogenesis. At 28 days after MCAO and treatment, the double IF staining of BrdU/NeuN and BrdU/GFAP dual-positive cells respectively indicating new neurons and glial cells emerged around ischemic regions, which was obvious in the BHD-NSC-EVs group and the number of BrdU/NeuN and BrdU/GFAP dual-positive cells in the BHD-NSC-EVs group was more than that in other groups (Figures 5B-c). Furthermore, we also assessed whether the BHD-NSC-EVs can promote micro-angiogenesis/capillary formation in ischemic regions of the brain, the markers of angiogenesis, vWF, was detected *via* IF staining cells. The IF staining cells demonstrated the upregulated expression of vWF around ischemic regions, the same as neuros and glial cells, the fluorescence intensity was more enhanced than that in other groups (Figures 5D-d). Meanwhile, the basis of mediating signals between neurons, the formation of neural synapses, was detected *via* markers of synaptic plasticity Postsynaptic Density-95 (PSD-95). The IF staining revealed the upregulated expression of PSD-95 around ischemic regions. In the BHD-NSC-EVs group, the fluorescence intensity of PSD-95 was more enhanced than that in other groups (Figures 5E-e). Taken together, these observations mentioned above verified that BHD-NSC-EVs facilitated neurogenesis and angiogenesis and promoted the neurovascular unit remodeling, which paved the way for neurological function recovery after stroke.

### 3.6 BHD-NSC-EVs promoted the proliferation and differentiation of NSCs *in vitro*

**FIGURE 6:**
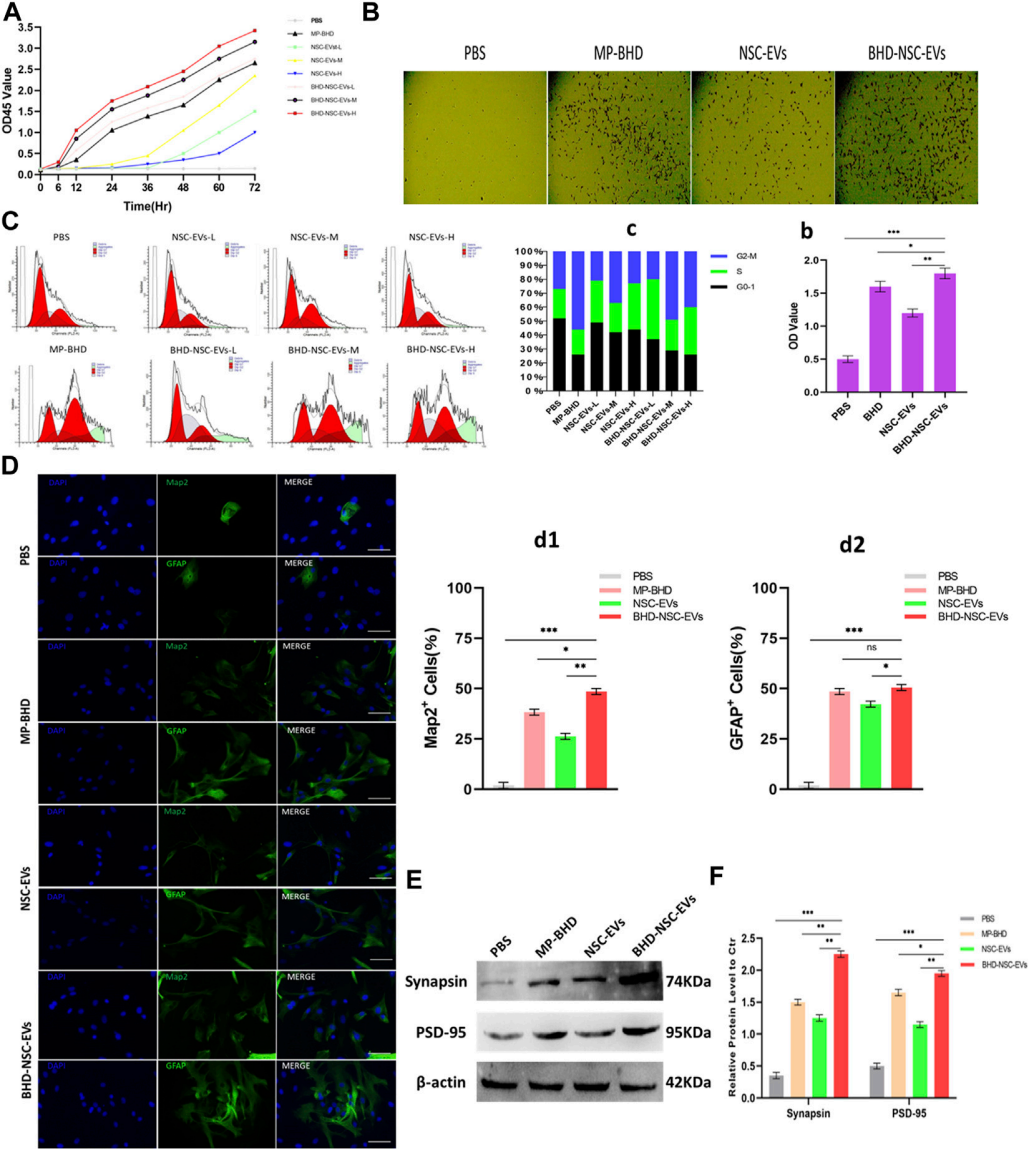
The impact of BHD-NSC-EVs on the neurogenetic activities of NSCs *in vitro*. **(A)** CCK-8 analysis was used to measure the effect of MP-BHD, NSC-EVs, BHD-NSC-EVs or PBS on the proliferation of NSCs, the mean OD45 value absorbance minus the blank value represented cell proliferation. NSC-EVs and BHD-NSC-EVs were added in wells from low to high dosage (L, 50 μg/mL; M, 100 μg/mL, H, 200 μg/mL). (*n* = 3/group), **(B-b)** Representative images of transwell assay for each treatment group to assess the impact on the neurogenetic activities of NSCs. *p < 0.05, **p < 0.01, ***p < 0.001, scale bar 100 μm in panel. **(C-c)** Flow cytometry of Cell cycle analysis was run to determine the effect of EVs from different dosage or MP-BHD or PBS on progression of NSC cell cycle stained with propidium iodide. EVs dosage: L, 7.5 μg/mL; M, 15 μg/mL, H, 30 μg/mL (*n* = 5/group). **(D-d)** Representative fluorescence staining with Map2 (green) or GFAP (green) and DAPI (blue) revealed differentiation into neuron and glial cells of NSCs treated with different inductions. (*n* = 5/group), *p < 0.05, **p < 0.01, ***p < 0.001, scale bar 100 μm in panel **(E-e)** Western blot analysis of the expression of marker of synapse-related factor Synapsin in differentiated NSCs. (*n* = 5/group), *p < 0.05, **p < 0.01, ***p < 0.001. All data are from three independent experiments and are presented as mean ± s.e.m,two-sided Student’s *t*-tests were used for comparisons **(A,b,c,d1,d2,e)**.

To clarify the direct functional role of BHD-NSC-EVs in the regulation of neurogenesis, the proliferation and differentiation of NSCs under the action of BHD-NSC-EVs were assessed. The CCK-8 analysis was used to measure the effect of MP-BHD, NSC-EVs, and BHD-NSC-EVs on the proliferation of NSCs.The above three types of intervention above can stimulate the proliferation of NSCs, especially the BHD-NSC-EVs treatment, which significantly promoted the proliferation of NSCs, with the effect increasing when the dosage of BHD-NSC-EVs was increased from low to high over time. In addition, MP-BHD exhibited similar properties to the low dosage BHD-NSC-EVs, while the effect of the NSC-EVs was weaker (Figure 6A). Following this, the impact of BHD-NSC-EVs on the neurogenetic activities of NSCs was assessed *via* a transwell assay, and was subsequently, compared with MP-BHD, NSC-EVs stimulation. Here, the BHD-NSC-EVs significantly promoted NSCs migration (Figures 6B-b). As cell proliferation is closely related to cell cycle progression, flow cytometry of cell cycle analysis was performed to determine the effect of the BHD-NSC-EVs on the progression of a NSC cell cycle stained with propidium iodide. The results indicated that MP-BHD, NSC-EVs, and BHD-NSC-EVs treatment were able to promote the cell cycle progression, but this effect of BHD-NSC-EVs treatment was more enhanced, characterized by the accumulation of cells in the G2/S phase (Figures 6C-c). To test the effects of BHD-NSC-EVs on the differentiation of NSCs, NSCs were co-cultured with MP-BHD, NSC-EVs and BHD-NSC-EVs for 1 week. The subsequent IF analysis suggested that the three types of intervention enhanced NSC differentiation, which was ascertained using the positive cells of MAP2 neuronal and GFAP cells versus the PBS controls. In the same field of view, the number of MAP2 neuronal and GFAP cells was higher in the BHD-NSC-EVs group than in other groups (Figures 6D-d1-2). In addition, another marker, the synapse-related factor, synapsin was assessed using the WB to ascertain the effect of BHD-NSC-EVs in terms of mediating signals between the neurons in cells. The results demonstrated that the neural synapses marker protein synapsin was present in the differentiated NSCs stimulated by MP-BHD, NSC-EVs, and BHD-NSC-EVs for 1 week. As with the previous results, the effect of the BHD-NSC-EVs treatment was more enhanced than that of the other treatments (Figures 6E-e).

Overall, the results indicated that BHD-NSC-EVs treatment augments the neurogenetic activities of NSCs, and significantly facilitates the proliferation and differentiation of NSCs compared with other treatments.

### 3.7 Involvement in miRNA profile of BHD-NSC-EVs by the mechanism for neurogenesis of BHD

**FIGURE 7:**
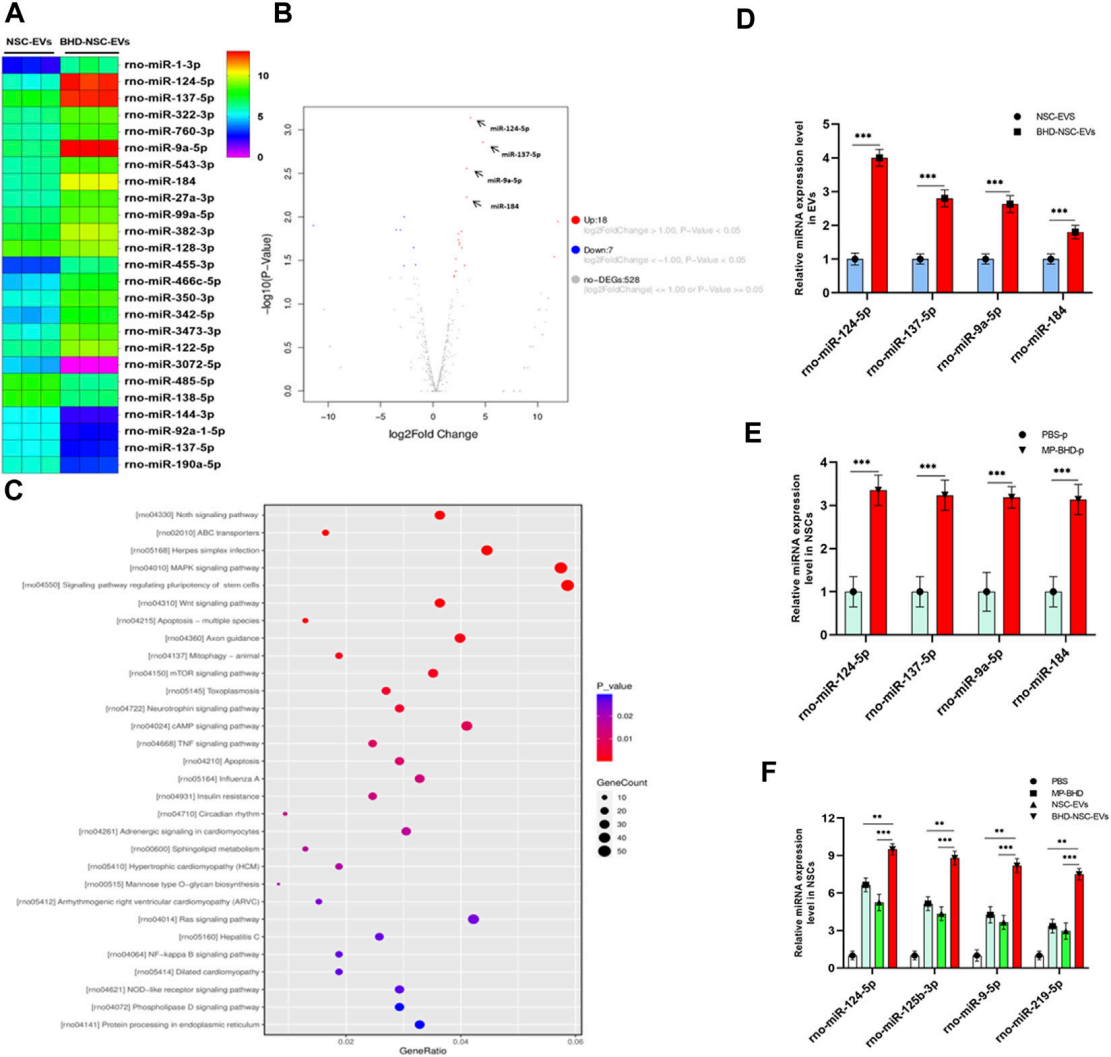
A mechanism for the neurogenesis of BHD-NSC-EVs by changing the miRNAs Profiles. **(A)** Heat map shows that 25 miRNAs exhibit Differential expression abundance in BHD-NSC-EVs and NSC-EVs. miR-124-5p, miR-9a-5p, miR-137-5p, and miR-184 are the top four of the upregulated miRNAs with markedly greater readcount. **(B)**Volcano plot analysis for the differentially miRNA expression in BHD-NSC-EVs vs. NSC-EVs. Red, significantly upregulated miRNAs; blue, significantly downregulated miRNAs; gray, no significant difference. Fold change >2 and *p* < 0.05 were considered significant. (*n* = 3/group). **(C)** KEGG pathway analysis of the target genes of the top four of the upregulated miRNAs encapsulated BHD-NSC-EVs. Top 30 enriched pathways are indicated. **(D)** qPCR analysis showed the relative miRNA expression level of the top 4 of the upregulated miRNAs encapsulated in BHD-NSC-EVs and NSC-EVs, (*n* = 5/group), Data were normalized to levels of U6, ***p < 0.001. **(E)** qPCR analysis showed the relative miRNA expression level of the top four of the upregulated miRNAs in NSCs after 12 h of MP-BHD or PBS treatment. (*n* = 5/group), Data were normalized to levels of *β*-Actin, ***p < 0.001. **(F)** qPCR analysis showed the relative miRNA expression level of the top four of the upregulated miRNAs in recipient NSCs after 12 h of BHD-NSC-EVs, NSC-EVs, MP-BHD or PBS treatment. (*n* = 5/group). Data were normalized to levels of *β*-Actin, *p < 0.05, **p < 0.01. ***p < 0.001. All data are from three independent experiments and are presented as mean ± s.e.m,two-sided Student’s *t*-tests were used for comparisons **(D–F)**.

As a form of nanovesicles, EVs are well known to encapsulate cargos, including proteins and nucleic acid, to exert a biological function. To further understand the potential mechanism through which BHD-NSC-EVs promote NSCs to proliferate and differentiate more significantly than NSC-EVs *in vitro* and *in vivo*, NGS of small RNAs was conducted to track the difference in the miRNA profiles of BHD-NSC-EVs and NSC-EVs. The sequencing samples of BHD-NSC-EVs and NSC-EVs were isolated from six samples (*n* = 3 in each group). First, non-coding RNA databases were established with exclusion of the contaminants, adaptors, low-quality reads, and reads smaller than 17 nt. In the remaining reads, the proportion of miRNAs isolated from the BHD-NSC-EVs and NSC-EVs was 25.20% ± 3.10% and 20.75% ± 2.5%, respectively, with the difference statistically significant (*p* < 0.05). Over 983 and 982 miRNAs were detected in the BHD-NSC-EVs and NSC-EVs, with 477 (48.5%) and 454 (46.2%) miRNAs existing stably from the three samples of BHD-NSC-EVs and NSC-EVs, respectively (Supplementary Figure S2). The overlapping and distinct miRNAs between the BHD-NSC-EVs and NSC-EVs, as well as a set of miRNAs that were differentially expressed, were identified. Compared with the miRNA expression in the NSC-EVs, the expression of 18 miRNAs in the BHD-NSC-EVs was upregulated, while seven miRNAs were downregulated. Among them, the top four upregulated miRNAs with the highest read counts were miR-124-5p, miR-9a-5p, miR-137-5p, and miR-184, which exhibited a significantly higher abundance in the BHD-NSC-EVs group than in the NSC-EVs group (Figures 7A, B). This result was verified through performing qRT-PCR analysis on the EVs (Figure 7C). Following this, Gene Ontology, and Kyoto Encyclopedia of Genes and Genomes analyses were performed to predict the target genes of the miRNAs with altered expression. Importantly, the top four miRNAs are associated with the regulation of neural development and cell biological behaviors of NSCs. Here, these miRNAs might interact with their target gene to exert specific biological functions, such as *Sox9, SCP1, BAF53a, BAF45a, REST* (miR-124-5p) and *TLX, FOXG1, REST, Her5, Her9* (miR-9a-5p), which can promote NSCs differentiation, as well as the *Ezh2* (miR-137-5p) and *Numb1* (miR-184) that can promote NSCs proliferation (according to NGS data analysis, not shown). The NGS results indicated that the Notch signaling pathway, the signaling pathway regulating the pluripotency of stem cells, axon guidance, and the MAPK signaling pathway might play an essential role in the proliferation and differentiation of NSCs (Figure 7D). To further verify the stimulation of MP-BHD on the top four miRNAs in NSCs, the cells stimulated with MP-BHD or PBS were harvested for qRT-PCR analysis. The relative expression levels of the top four miRNAs in the NSCs stimulated with BHD-NSC-EVs were markedly higher than that those stimulated by PBS (Figure 7E). Meanwhile, qRT-PCR transfection analysis results indicated that the relative expression levels of the top four miRNAs in the NSCs, stimulated with BHD-NSC-EVs were markedly higher than those stimulated by the other treatments (Figure 7F).

Collectively, these results indicate that MP-BHD induces NSCs to secrete BHD-NSC-EVs encapsulating altered miRNAs, which are associated with the regulation of neural development and the biological behaviors of NSCs. This could be the potential mechanism behind the enhanced neurogenesis *via* BHD-NSC-EVs *in vitro* and *in vivo*.

## 4 Discussion

**FIGURE 8:**
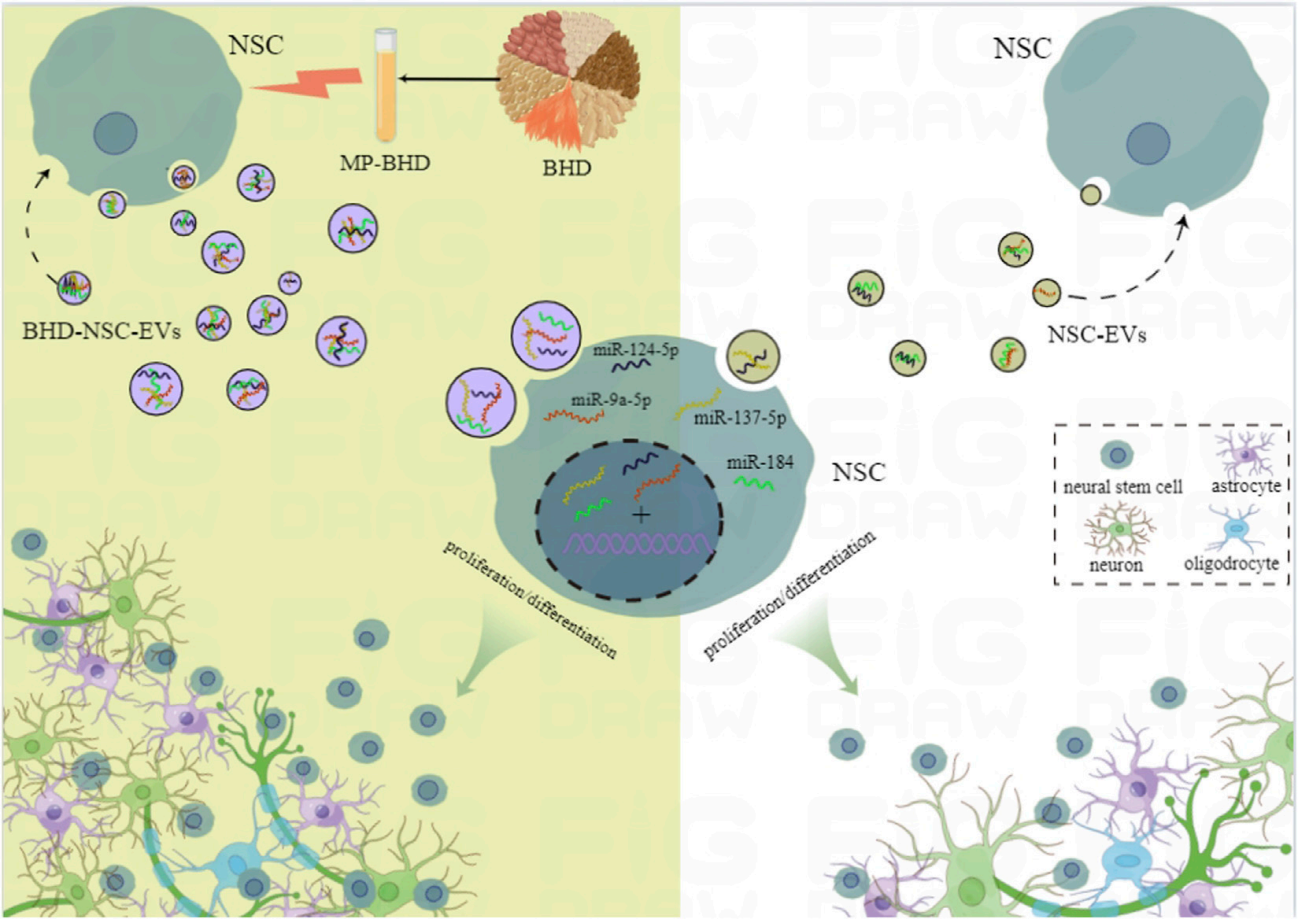
Schematic diagram of the potential mechanism of Buyang Huanwu Decoction for the neurogenesis in our study. The effective components of Buyang Huanwu Decoction contained in medicated plasma promoted the large-scale generation of extracellular vesicles derived from NSCs and recompiled the encapsulated miRNAs profiles. This kind of BHD-NSC-EVs significantly enhanced the proliferation and differentiation of NSCs and effectively accelerated neurological function recovery after ischemic stroke. MP-BHD, medicated plasma of Buyang Huanwu Decoction; NSCs, neural stem cells; NSC-EVs, extracellular vesicles derived from neural stem cells; BHD-NSC-EVs, extracellular vesicles derived from medicated plasma of Buyang Huanwu Decoction-preconditioned neural stem cells.

In this study, based on the concrete clinical efficacy of BHD on survivors of an ischemic stroke, MP-BHD was prepared to precondition primary NSCs and isolate BHD-NSC-EVs. The therapeutic effect of BHD-NSC-EVs on the acquired neurological impairment following an ischemic stroke was demonstrated through *in vivo* and *in vitro* experiments. The data suggested that the MP-BHD promoted the NSCs to secrete the sufficient amount of EVs required for therapeutical effect. The BHD-NSC-EVs could enhance neurogenesis and rehabilitate the neurological function of MCAO model rats through facilitating the neurovascular unit remodeling involved in the proliferation and differentiation of NSCs. The effects of BHD-NSC-EVs were consistent with the BHD used *in vivo* and the MP-BHD used *in vitro*. Furthermore, the BHD-NSC-EVs had a more significant effect than BHD or NSC-EVs alone. Meanwhile, the NGS results for the miRNA profiles in the EVs suggested that the BHD-NSC-EVs induced the neurogenetic activities of NSCs in terms of proliferation and differentiation by using their cargos, which mainly included miR-124-5p, miR-9a-5p, miR-137-5p, and miR-184, with these more abundant in the BHD-NSC-EVs group than in the NSC-EVs group. These cargos are associated with the regulation of neural development and the biological behaviors of NSCs, which is the possible reason for the enhanced neurogenesis and rehabilitating the neurological function by BHD-NSC-EVs compared to other treatments. (Figure 8).

In traditional Chinese medicine (TCM) theory, the main causes of ischemic stroke are Qi deficiency and blood stasis (Zhang et al., 2021b; Zheng et al., 2021). Therefore, the main method for treating an ischemic stroke involves nourishing the Qi and activating the blood circulation. The BHD potion is a classic TCM formulation and has long been used for therapy targeting hemiplegia following a stroke in Chinese medicinal practice. The main effect of BHD has been identified as regulatory, i.e., Qi-tonifying and stasis-eliminating, or, put another way, enhancing the blood circulation, activating the physiological functions of the body, and invigorating the body through energy meridians. In the present study, both the BHD used in the MCAO model rats to reinstate the limb motor function and the MP-BHD used with cultured primary NSCs to proliferate, differentiate, and secrete abundant EVs reflected the activation effect of BHD, as well as the impact on angiogenesis. Furthermore, the formation of neural synapses reflected the efficacy of BHD in terms of enhancing the blood circulation and mediating the signals between neurons.

Chinese herbs and their formulations contain complex components. For the use of TCM in *in-vitro* experiments, MP or plasma pharmacology has been developed and generally accepted as part of a standardized experimental method (Ge et al., 2010; Lin et al., 2018). Here, MP-BHD presents an *in-vitro* model of BHD treatment and has the same efficacy as BHD, meaning the stimulation of MP-BHD on cultured primary NSCs *in vitro* equates to the drug action of BHD *in vivo*. In the present study, BHD accelerated neurological impairment recovery of MCAO model rats may through its effective composition which were absorbed in plasma and acted on intrinsic NSCs as a therapeutic role.

In addition to NSC transplantation, another way to use NSCs for treatment involves the activation of intrinsic stem cells. However, the quantity of the activated intrinsic NSCs for newly born neurons is too low to restore the neurologic functions. For example, in the MCAO model rats, only 0.2% of the dead striatal neurons could be replaced by newly born neurons. Thus, to enable the use of intrinsic NSC sources for therapeutic purposes, appropriate interventions must be added to enhance the proliferation, survival, and neuronal maturation of intrinsic NSCs and their progeny. Both the oral administration of BHD for the treatment of hemiplegia following a stroke and the intragastric administration in the MCAO model rats may have been involved in the activation of intrinsic NSCs.

EVs play the important roles of cell micro-communication and bioinformation transportation in stem cells, and their internal contents determine the biological functioning (EL Andaloussi et al., 2013). Derived from stem cells exhibit stem-cell-like pro-regenerative properties but are not live cells, meaning direct treatment with EVs may prevent many of the adverse effects of stem cell transplantation therapy. The low efficacy of NSC therapy, as demonstrated by the low survival rate, can be overcome by adopting NSC-EVs therapy. Studies have demonstrated that EVs derived from mesenchymal stem cells (MSCs) could mimic the capability of MSCs to modulate the activity of a wide range of immune cells (Delavogia et al., 2022), while umbilical cord-derived MSC EVs have the potential to prevent liver I/R injury by reducing CD154 expression (Zheng et al., 2020). Meanwhile, the application of NSC-derived small EVs treatment has the potential to reduce neuronal apoptosis, inhibit neuroinflammation, and promote functional recovery in spinal cord injury model rats (Rong et al., 2019). Human urine-derived stem cell (USC) exosome treatment could protect against ischemia/reperfusion-injury-induced renal damage, the effect of which is equal to treatment with USCs (Li et al., 2020). The exosomes isolated from the pre-differentiated human MSCs can induce osteogenic differentiation to achieve cell-free bone regeneration (Zhai et al., 2020). In the treatment process, the EVs derived from stem cells play a key role, presenting a suitable alternative to cell-based therapies. In the present study, the EVs secreted by NSCs in a physiological state facilitated neurogenesis and rehabilitated the neurological functioning, consistent with the BHD and MP-BHD, demonstrating the therapeutic effects of EVs or EXOs as a new therapeutic treatment for ischemic strokes.

In addition to the yield of EVs, the signaling cargos encapsulated in EVs are essential to their efficacy (Saraiva et al., 2017). This can vary from different pathological conditions, such as oxidative stress, heat stress, and inflammatory stimulation, and might cause a completely reversed fate of the target cell (Yang et al., 2020). For example, EVs derived from MSCs cultured in hypoxia (3% oxygen) (hypoxic MSC-EVs) have a higher abundance of miR-612 than in normoxic conditions and can significantly enhance the angiogenesis for ischemic diseases (Ge et al., 2021). Under oxidative stress, the cardiac progenitor cells secrete more EXOs containing miR-21 compared with under normal conditions, which could protect myocardial cells against oxidative-stress-related apoptosis in ischemic cardiac diseases (Xiao et al., 2016). Meanwhile, pro-inflammatory factor interferon gamma (IFN-γ) can significantly alter the ability of human NSC-derived EXOs (IFN-γ-hNSC-EXO) better than hNSC-EXO, and further induce therapeutic effects in ischemic stroke model rats through the miRNAs contained within, including miR-206, miR-133a-3p, and miR-3656 (Zhang et al., 2020). In present study, the MP-BHD promoted the NSCs to secrete a sufficient amount of EV loading altered miRNAs, which can induce degradation or translational inhibition of target mRNA by complementation and pairing with the target mRNA, thus inducing the function of regulating gene expression. The top four upregulated miRNAs, namely, miR-124-5p, miR-9a-5p, miR-137-5p, and miR-184 in BHD-NSC-EVs are essential to the efficacy of NSC-EVs. In fact, miR-124-5p is the most abundant miRNA in NSCs and can directly target the small C-terminal domain phosphatase 1 (an anti-neural factor) 3′ untranslated region (SCP1-3′UTR) to suppress SCP1 expression and induce neurogenesis, also playing a key role in the differentiation of progenitor cells and mature neurons by regulating an intricate network of NSC-specific alternative splicing (Shi et al., 2016). Meanwhile, miR-9a-5p is expressed specifically in the neurogenic regions of the brain and can negatively regulate NSC proliferation and accelerate neural differentiation by forming a negative regulatory loop with the nuclear receptor, TLX (Madelaine et al., 2017). The miRNA, miR-137-5p, involves co-regulation by SOX2 (a core transcription factor in stem cells) and could repress the transcription of Ezh2 (a histone methyltransferase and Polycomb group protein), resulting in the reduction of histone H3 trimethyl lysine 27, thereby modulating the proliferation and differentiation of NSCs *in vitro* and *in vivo* (Mahmoudi and Cairns, 2017). Finally, miR-184 regulates the expression of Numblike (a known regulator of brain development) by binding to its 3′UTR to control the balance between the proliferation and differentiation of NSCs, with high levels of miR-184 promoting NSC proliferation and inhibiting differentiation both *in vitro* and *in vivo* (Liu et al., 2010)*.*

Overall, the study results indicated that in replacing BHD, NSCs or NSC-EVs, BHD-NSC-EVs can serve as a highly efficient therapeutic agent for enhancing neurogenesis and improving the neurological impairment induced by an ischemic stroke.

This study involves a number of limitations. First, the main active components of BHD, the main factors in the pharmacological effect, were not identified. In future studies, the ultraperformance liquid chromatography-tandem mass spectrometry method and network pharmacological analysis can be used to determine the main active components of BHD and their potential targets. Furthermore, the mechanisms through which NSCs secrete large amounts of EVs in response to the main active components of BHD can be detected in NSCs using single-cell sequencing technology. In addition, while the EV RNA-sequencing suggested that the mechanism behind the neurogenesis of BHD-NSC-EVs relates to the alteration of their miRNAs profiles, experiments for verifying the mechanism of miRNAs and the signaling pathway *in vivo* and *in vitro* were not conducted. In future studies, miRNA mimics and inhibitors and perturbation-of-function approaches using specific antagonists and agonists could be adopted to explore the miRNA function, while a dual luciferase reporting system could be used to determine the interaction between miRNAs and their target genes.

Overall, the findings of this study demonstrate that BHD-NSC-EVs significantly enhance neurogenesis and effectively accelerate the neurological function recovery of MCAO model rats. In this process, MP-BHD promoted the large-scale generation of BHD-NSC-EVs and recompiled the encapsulated miRNAs profiles, miR-124-5p, miR-9a-5p, miR-137-5p, and miR-184. These were the top four upregulated miRNAs with the highest read counts, and are all associated with neurogenesis and play an essential role in the process of the regulation of neural development and the cell biological behaviors of NSCs. In replacing BHD, NSCs, or NSC-EVs, BHD-NSC-EVs can be utilized as a part of a novel therapeutic nano-delivery system for ischemic stroke therapy. Based on the clinical efficacy of TCM, the preconditioning of NSC-derived EVs *via* the MP of Chinese herbs, presents a newly promising therapeutic strategy for neurological diseases.

TCM herbs-derived medicated plasma, as a method to preserve and enhance the effectiveness of complex TCM herbs *in vitro*, may provide a new idea for the development of a non-drug, non-cellular nanotherapy.

## Data Availability

The raw data supporting the conclusions of this article will be made available by the authors, without undue reservation. However, due to Southern Medical University has the right to keep the data confidential, the data are not uploaded to the public database. For further enquiries, please contact the corresponding author (LC, neuro_clk@hotmail.com).

## Glossary

**ALT**: alanine aminotransferase
**AST**: aspartate aminotransferase
**BCA**: bicinchoninic acid assay
**BHD**: Buyang Huanwu Decoction
**BHD-NSC-EVs**: MP-BHD to precondition primary NSCs and isolated EVs
**BuN**: blood urea nitrogen
**CCA**: common carotid artery
**DAPI**: 4′, 6-diamidino-2-phenylindole
**DiR**: 1,1-dioctadecyl-3,3,3,3-tetramethy-lindotricarbocyanine iodide
**DMEM**: dulbecco’s modified eagle medium
**ECA**: external carotid artery
**EVs**: extracellular vehicles
**GO**: Gene Ontology
**ICA**: internal carotid artery
**IF**: immunofluorescence
**IFN-γ**: pro-inflammatory factor interferon gamma
**IVIS**: Living Imaging System
**KEGG**: Kyoto Encyclopedia of Genes and Genomes pathway
**LSIS**: laser speckle imaging system
**MCAO**: middle cerebral artery occlusion
**MP-BHD**: medicated plasma of Buyang Huanwu Decoction
**MRI**: magnetic resonance imaging
**MSCs**: mesenchymal stem cells
**NSCs**: Neural stem cells
**NTA**: nanoparticle tracking analysis
**OD value**: absorbance
**PBS**: phosphate buffer saline
**PFA**: paraformaldehyde
**PTCM**: traditional Chinese medicine
**SCP1-3′UTR**: small C-terminal domain phosphatase 1, 3′untranslated region
**SD-95**: synaptic plasticity Postsynaptic Density-95
**SDS-PAGE**: Sodium Dodecyl Sulfate Polyacrylamide Gel Electrophoresis
**SOX2**: sex determining region Y-box 2
**TEM**: transmission electron microscopy
**TMM**: trimmed mean of M-values
**TTC**: 2,3,5-triphenyl tetrazolium chloride
**USC**: urine-derived stem cell
**vWF**: von Willebrand factor
**WB**: western blotting
