# Supplementary material for: Extracellular vesicles from medicated plasma of Buyang Huanwu decoction-preconditioned neural stem cells accelerate neurological recovery following ischemic stroke
Source: Front Cell Dev Biol. 2023 Mar 1;11:1096329. doi: 10.3389/fcell.2023.1096329 (PMC10014837; doi:10.3389/fcell.2023.1096329)
Supplement: Supplementary file 1 [file DataSheet1.DOCX]

**supplementary files**


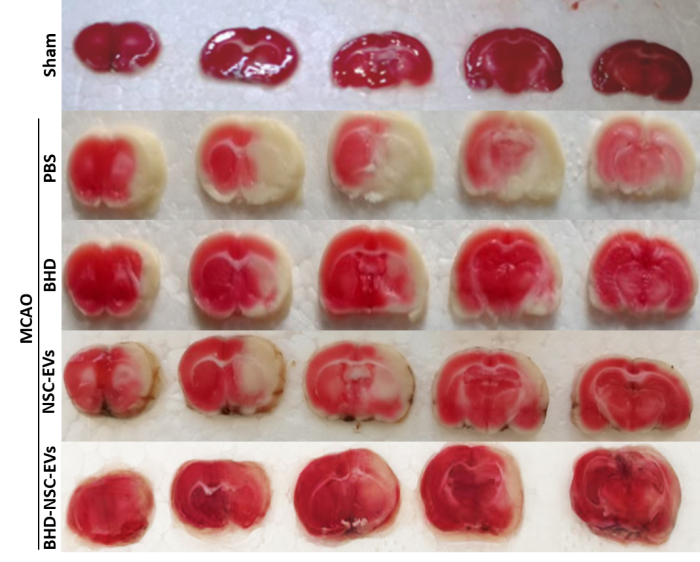


**Fig.S1:** RepresentativeTTC staining images showed the effect of MCAO surgery and the lesion area of MCAO changed in the treatment group 4 weeks post MCAO onset, the therapeutic efficacy of BHD-NSC-EVs.was more significant than others.


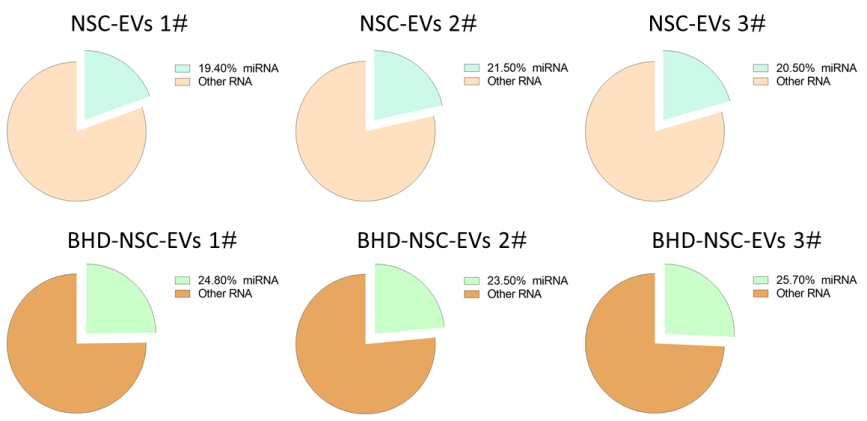


**A**


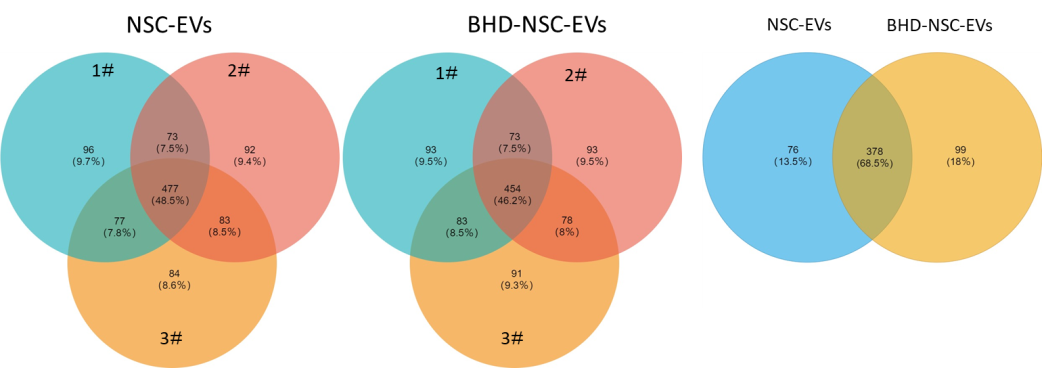


**B**

**Fig.S2:**The comparison for the miRNA profiles of BHD-NSC-EVs and NSC-EVs.(A) The proportion of miRNAs isolated from BHD-NSC-EVs and NSC-EVs.(B) total miRNAs were simultaneously identified both in BHD-NSC-EVs and NSC-EVs. The number of the overlapping and the unique miRNA between the two groups were distinguished.


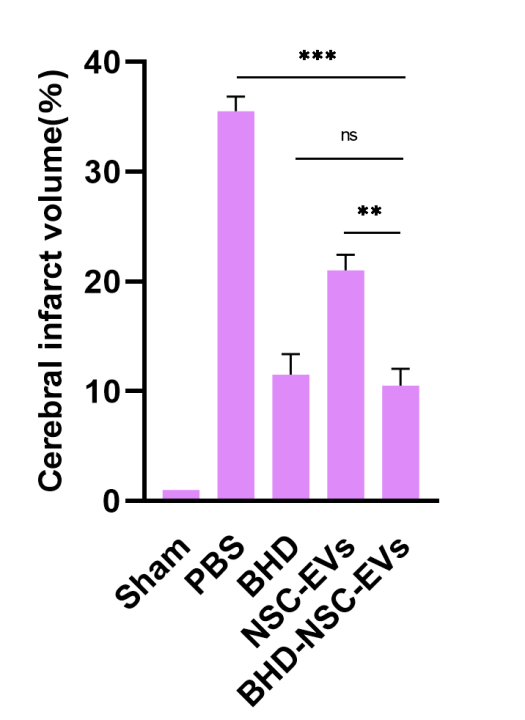


**Fig.S5a:** The quality of immunocytochemistry images, (n=7), ns no significant, **P < 0.01, ***P < 0.001, All data are from three independent experiments and are presented as mean ± s.e.m,two-sided Student’s t-tests were used for comparisons.


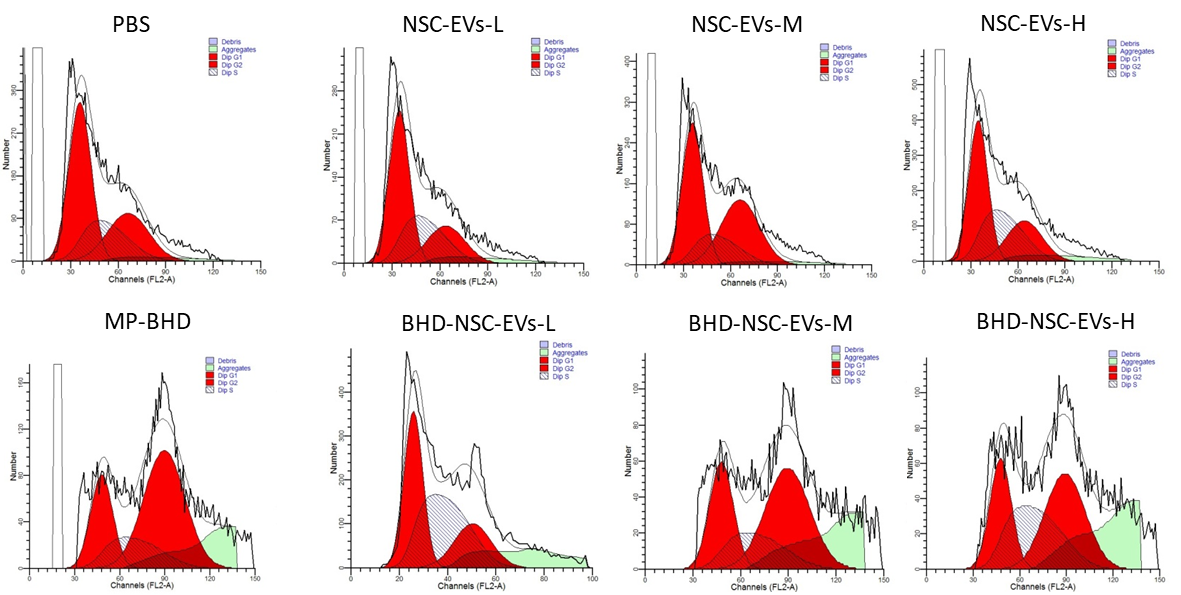


**Fig.S6C**: Flow cytometry of Cell cycle analysis was run to determine the effect of EVs from different dosage or MP-BHD or PBS on progression of NSC cell cycle stained with propidium iodide.
